# Preterm Birth Increases Susceptibility to Hyperglycemia‐Induced Kidney Injury With Sex‐Specific Differences in Structural and Molecular Responses

**DOI:** 10.1002/edm2.70223

**Published:** 2026-04-20

**Authors:** Rachel K. Dailey, Aleksandra Cwiek, Logan C. Hamil, Sage Timberline, Ayyappa Kumar Sista Kameshwar, Masako Suzuki, Jaya Isaac, Kimberly deRonde, Mark Conaway, Kevin M. Bennett, Edwin J. Baldelomar, Matthew R. Hoch, Kimberly J. Reidy, Jennifer R. Charlton

**Affiliations:** ^1^ Division of Nephrology, Department of Pediatrics University of Virginia Charlottesville Virginia USA; ^2^ Department of Cell Biology University of Virginia Charlottesville Virginia USA; ^3^ University of Virginia Charlottesville Virginia USA; ^4^ Division of Critical Care Phoenix Children's Hospital Phoenix Arizona USA; ^5^ Department of Nutrition Texas A&M University College Station Texas USA; ^6^ Division of Pediatric Nephrology, Department of Pediatrics Children's Hospital at Montefiore Einstein Bronx New York USA; ^7^ University of Virginia Health System Charlottesville Virginia USA; ^8^ Division of Translational Research and Applied Statistics, Department of Public Health Sciences, University of Virginia School of Medicine University of Virginia Charlottesville Virginia USA; ^9^ Mallinckrodt Institute of Radiology, Washington University School of Medicine St. Louis Missouri USA; ^10^ Department of Biomedical Engineering University of Virginia Charlottesville Virginia USA

**Keywords:** cationic ferritin enhanced‐MRI, chronic kidney disease, diabetic kidney disease, endothelial‐podocyte crosstalk, preterm birth, streptozotocin

## Abstract

**Background:**

Preterm birth increases the long‐term risk of diabetes and chronic kidney disease (CKD), yet its impact on diabetic kidney disease (DKD) is unclear. We previously showed that male preterm mice with diabetes develop early features of DKD, including reduced podocyte density, decreased renin expression, activation of angiogenesis pathways, and impaired endothelial–podocyte signaling. Here, we examine whether preterm birth similarly accelerates DKD progression in female mice and compare structural and transcriptomic outcomes in the females to the prior male cohort to assess sex‐specific differences.

**Methods:**

Preterm mice were delivered by Caesarean section at 19 days post conception (dpc) and term mice at 20 dpc. Hyperglycemia was induced at 6 weeks with streptozotocin to generate term‐diabetic (T‐D) and preterm‐diabetic (PT‐D) groups; controls were term‐nondiabetic (T‐ND) and preterm‐nondiabetic (PT‐ND). Body weight and glucose were monitored, and kidneys were analyzed at 18 weeks using histologic, stereologic, imaging, and transcriptomic methods.

**Results:**

Compared with T‐ND females, PT‐D females showed higher albuminuria, more atubular glomeruli, reduced proximal tubule (PT) fraction, and pro‐fibrotic gene activation. Compared to T‐D females, PT‐D females had higher blood urea nitrogen (BUN) levels and reduced PT fraction. This was associated with activation of pathways associated with the vasculature and suppression of mitochondrial metabolism gene pathways, along with Notch signalling alterations. Sex differences included a lower PT fraction in preterm females than males and fewer atubular glomeruli in PT‐D females than PT‐D males. Renin expression was lower in PT‐D than T‐D in males only. When comparing PT‐D to T‐D across sexes, 582 differentially expressed genes were unique to females. Notch signalling was upregulated in both male and female PT‐D mice compared to T‐D.

**Conclusion:**

Preterm birth increases susceptibility to kidney injury in females after exposure to hyperglycemia. However, preterm females with hyperglycemia are more resistant to kidney damage than males.

## Graphical abstract

Preterm mice were delivered at 19 dpc and rendered diabetic with STZ at 6 weeks; kidneys were analysed at 18 weeks. PT‐D females showed increased atubular glomeruli, reduced proximal tubule fraction, higher albuminuria and BUN, and dysregulated fibrotic, vascular, mitochondrial, and Notch pathways. Sex‐specific differences included greater injury and renin alterations in males.

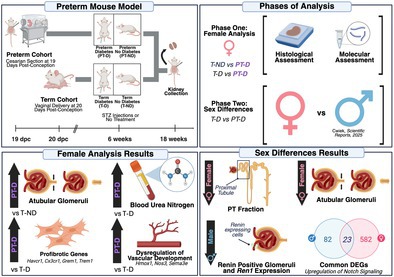

## 1 Introduction

Diabetes affects nearly 10% of adults in the United States and is becoming increasingly prevalent across the world [1]. Diabetic kidney disease (DKD) is a common complication of diabetes. DKD is the leading cause of chronic kidney disease (CKD) in adults and accelerates the progression to end stage kidney disease (ESKD). In addition to accelerating kidney decline, patients with DKD are at an increased risk for cardiovascular‐related illness and death and face significant psychosocial and financial burdens [2]. There is an urgent need to identify factors that influence DKD risk to facilitate the development of interventions to slow or halt its progression.

Preterm birth has been independently associated with increased risk of both diabetes and CKD [3, 4] and has the potential to play a role in the progression of DKD. Preterm birth (< 37th week of gestation) occurs in approximately one in 10 infants in the United States [5]. Preterm neonates likely have a truncated window of nephrogenesis, a critical period during which the lifetime supply of nephrons is formed [6, 7, 8]. Autopsy studies have shown a higher proportion of morphologically abnormal glomeruli in human preterm neonates, suggesting abnormal nephrogenesis [9]. This truncation and abnormal development may explain in part why neonates of extremely low gestational age have a significant risk for CKD early in life [4, 10]. Moreover, epidemiologic studies show that preterm birth also increases the likelihood of developing diabetes in adulthood [3, 11, 12], compounding susceptibility to CKD. The mechanistic basis for the association between preterm birth and CKD and diabetes lies in the concept of developmental programming, whereby in utero insults cause lasting alterations to fetal growth trajectories, endocrine function, and metabolic pathways. Such exposures have the potential to impair the offspring's pancreatic β‐cell development or reduce β‐cell mass, or reduce and impair insulin secretion, leading to a type 1 diabetic phenotype. Alternatively, offspring subjected to adverse maternal environments can develop insulin resistance, with altered adipocyte development and skeletal muscle glucose uptake, exhibiting a type 2 diabetic phenotype [13]. Although the associations between preterm birth and both CKD and diabetes are increasingly understood, the role of preterm birth as an upstream driver of DKD has not been well studied.

Biologic sex is an additional modifier of the risk of DKD. Although the role of preterm birth as an upstream factor for DKD is understudied, evidence suggests that sex‐specific differences may influence outcomes in those born preterm [14]. Sexual dimorphisms have been clearly identified in adulthood CKD. While women are more likely to be diagnosed with CKD, men are more likely to experience a faster and more progressive decline in kidney function [15]. In general, women appear to be protected against CKD before menopause. After menopause, women have a greater risk of progressive kidney disease than men [16]. Oestrogen may provide protection to the kidney through mechanisms such as inhibition of the renin‐angiotensin system and reduction of injury to mesangial cells and podocytes in the glomerulus [17]. Understanding the mechanisms underlying this protection in females is important because they may inform strategies to develop therapeutic targets to induce similar renoprotective effects in males.

This study follows on recent work investigating the effects of preterm birth on DKD development in male mice. In Cwiek et al., hyperglycemia was induced using streptozotocin in adult male mice that were born preterm to investigate the effects of preterm birth on structural and transcriptomic alterations in diabetic kidneys [18]. Due to known sex differences in models of streptozotocin‐induced DKD, mice were stratified by sex a priori [19, 20]. After exposure to hyperglycemia, the preterm male mice had histologic alterations compared to term male mice that suggested that preterm birth is a risk factor in DKD [18]. These alterations included reduced podocyte density, lower proximal tubule area, and greater disruption of the tubular connection to the glomerulus resulting in more atubular glomeruli. Preterm males with hyperglycemia also had transcriptomic differences, exhibiting lower expression of the gene encoding renin (*Ren1*) and increased extracellular matrix gene expression. Additionally, cell–cell communication was altered in the preterm diabetic compared to term diabetic males, with diminished crosstalk between endothelial cells and podocytes, which may have contributed to the loss of podocytes.

Building on our group's findings in the male cohort of mice, the goal of this study was to evaluate the impact of preterm birth on kidney injury in female mice exposed to hyperglycemia and to compare outcomes between sexes. We characterized structural and transcriptomic changes in kidneys of female mice subjected to both preterm birth and streptozotocin‐induced hyperglycemia. Comparisons were made with term nondiabetic females to assess the combined effects of preterm birth and hyperglycemia, and with term diabetic females to isolate the contribution of preterm birth in the diabetic state. We hypothesized that preterm diabetic females would exhibit greater injury than term nondiabetic females and more severe changes than term diabetic females. We further anticipated that females would show fewer or less pronounced alterations than males, reflecting sex‐specific protection. This study aims to clarify how preterm birth and hyperglycemia interact to influence DKD susceptibility, and to define the contribution of early birth and sex to diabetic kidney disease progression.

## 2 Materials and Methods

Reagents and resources used in these experiments are shown in Table S1.

### 2.1 Preterm Mouse Model

This study was performed in accordance with the recommendations in the Guide for the Care and Use of Laboratory Animals of the National Institutes of Health. All animal experiments were approved by the Institutional Animal Care and Use Committee of the University of Virginia and reported following ARRIVE (Animal Research: Reporting of In Vivo Experiments) guidelines.

CD‐1 mice from Charles River Laboratory (Wilmington, MA, USA) were used to establish the preterm mouse model as previously published [18, 21, 22]. In brief, timed pregnant dams were placed overnight with a breeder and pregnancy was confirmed by the presence of a vaginal plug, marking the first day post conception (dpc). At 19 dpc, pregnant dams were euthanized via cervical dislocation. Preterm pups were delivered by Caesarean section, resuscitated, and placed with foster mothers. Acepromazine maleate (AceproJect 10 mg/mL, final concentration 75 mg/mL; Henry Schein Animal Health) was mixed in the drinking water for 24 h before and 24 h after fostering (total 48 h) to reduce the risk of preterm pup mortality from neglect or cannibalization by foster mothers. A second cohort of timed pregnant dams gave birth to their pups naturally at full term (20 ± 0.25 dpc). Term pups remained with their biological mothers. All mice were weaned at 3 weeks of age. At 6 weeks of age, half of preterm and term mice were randomly selected to be treated with streptozotocin (STZ) and litter mates served as term nondiabetic controls (T‐ND).

**FIGURE 1:**
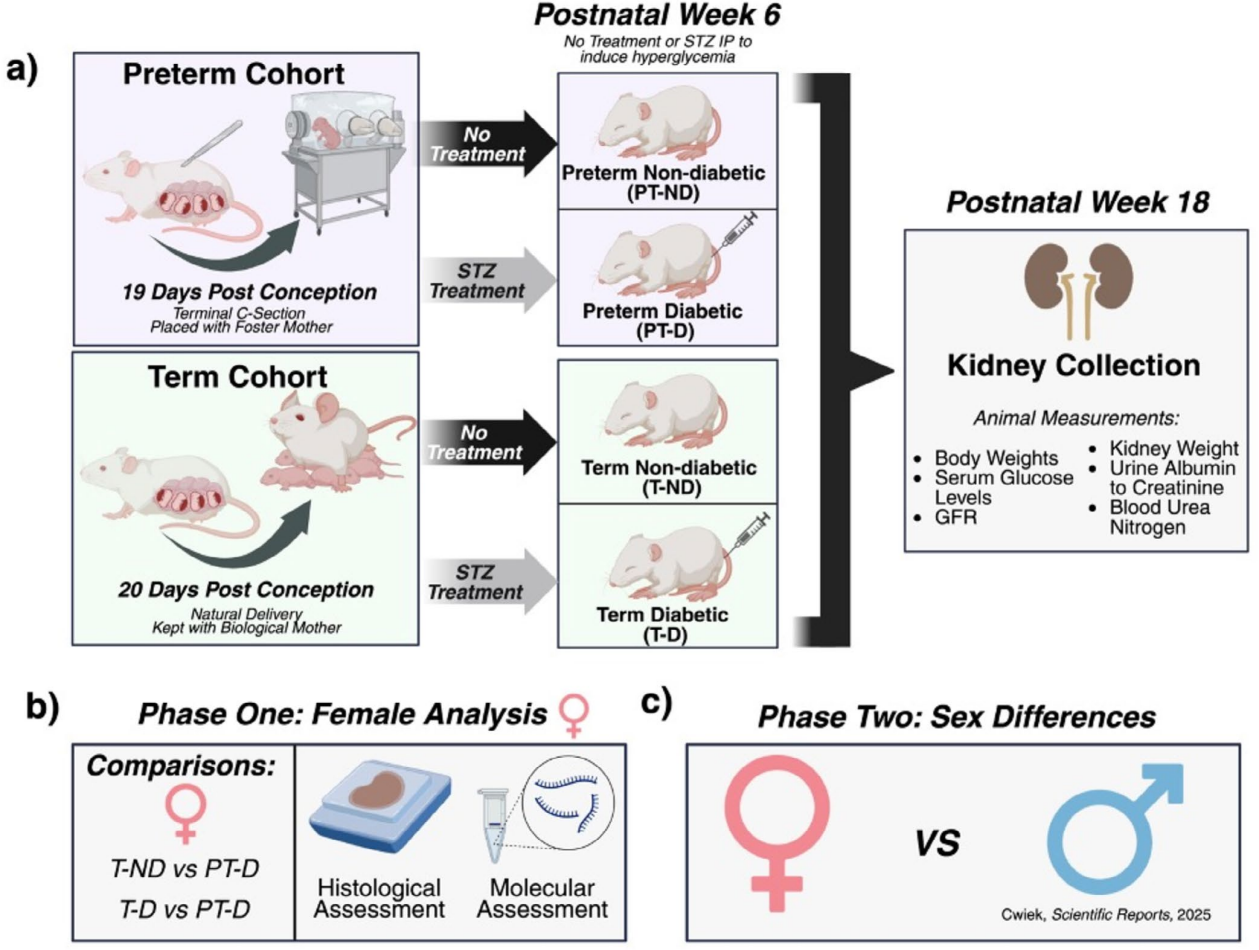
Establishment of a preterm mouse model with hyperglycemia. (a) Preterm mice were delivered via terminal C‐section 1 day before full term at 19 days post conception. Term mice were born vaginally at 20 days post conception. STZ was administered at 6 weeks to induce hyperglycemia, and mice were euthanized at 18 weeks. (b) The combined effect of preterm birth and hyperglycemia on structural and molecular alterations in the kidney was assessed through the comparison of preterm diabetic (PT‐D) and term non‐diabetic (T‐ND) females. (c) The effect of hyperglycemia on those born preterm was assessed by comparing preterm diabetic (PT‐D) and term diabetic (T‐D) females. Characterization of the male mice in this model has been previously published in Cwiek et al. [18]. Panels a, b, & c were created in BioRender.com.

Body weights (BW) were measured weekly after weaning. At 18 weeks of age, glomerular filtration rate (GFR) was measured, urine was collected, and mice received cationic ferritin injections (CF; 5.75 mg/100 g body weight) for imaging, as previously published [18, 23]. Ninety minutes after the second CF injection, mice were sedated with intraperitoneal (IP) tribromoethanol and underwent intracardiac perfusion with normal saline followed by 4% formalin. To preserve RNA integrity, the left renal pedicle was clamped prior to formalin perfusion, preventing formalin exposure to the left kidney. Kidneys were collected, weighed, processed for histological analysis, and total RNA was extracted for bulk mRNA sequencing (RNA‐Seq). The experimental design is illustrated in Figure 1a.

### 2.2 Induction of Hyperglycemia

At 6 weeks, female mice were assigned to one of the four experimental cohorts: term nondiabetic (T‐ND; *n* = 6), preterm nondiabetic (PT‐ND; *n* = 4), term diabetic (T‐D; *n* = 6), preterm diabetic (PT‐D; *n* = 5). The 6‐week timepoint, corresponding to early adulthood in mice, was selected to induce hyperglycemia based on previous studies [24] and to model the diabetes often seen in preterm patients during young adulthood [25].

Streptozotocin (STZ, Zanosar Teva Pharmaceuticals, Irvine, CA) injections were administered as previously described [20]. Hyperglycemia was induced using the low‐dose STZ protocol (IP injections at 50 mg/kg for 5 consecutive days) following a fasting period, as recommended by the Animal Models of Diabetic Complications Consortium. The low‐dose STZ protocol was followed to avoid the high mortality rates reported with a high‐dose STZ protocol [26]. Baseline plasma glucose levels prior to STZ treatment were measured at 6 weeks of age. Plasma glucose levels were assessed at one and 6 weeks after STZ treatment, and before euthanasia at 18 weeks. As recommended by the Animal Models of Diabetic Complications Consortium, mice with glucose concentrations < 300 mg/dL 6 weeks after STZ exposure underwent a second round of STZ injection using the same low‐dose STZ protocol (T‐D, *n* = 0; PT‐D, *n* = 3).

### 2.3 Glomerular Filtration Rate (GFR)

GFR was measured using a MediBeacon (Mannheim, Germany) transcutaneous fluorescent detection device, as previously published [18, 23]. At 18 weeks of age (T‐ND, *n* = 4; PT‐ND, *n* = 3; T‐D, *n* = 3; PT‐D, *n* = 4), fluorescein isothiocyanate (FITC)‐labelled sinistrin (7.5 mg/100 g BW) was administered via tail vein injection. Elimination kinetics curves were generated using MPD Studio software (Mannheim Pharma and Diagnostics, Amtsgericht Mannheim, Germany), and GFR was calculated as previously described [18, 23].

### 2.4 Urine Albuminuria and Blood Urea Nitrogen (BUN)

Urine samples were collected from mice by bladder massage or isolation in a plastic device prior to euthanasia (T‐ND, *n* = 5; PT‐ND, *n* = 4; T‐D, *n* = 6; PT‐D, *n* = 5). The samples were analysed for albumin and creatinine concentration to calculate the urinary albumin to creatinine ratio (ACR). ACR analysis was performed using the Albuwell M Mouse Albumin ELISA Kit (Ethos Biosciences, Logan Township, NJ, USA) and the DetectX Urinary Creatinine Detection Kit (K002‐H; Arbor Assays, Ann Arbor, MI, USA), following the manufacturers' protocols. BUN analysis was performed using a DetectX Urea Nitrogen (BUN) Colorimetric Detection Kit (Cat# K024‐H1; Arbor Assays, Washtenaw County, MI, USA), according to the manufacturer's instructions. Samples were stored at −20°C from collection until analysis.

### 2.5 Immunohistochemistry and Histology

Kidneys were immersion‐fixed in 10% buffered formalin, embedded in paraffin, and sectioned at 5 μm thickness. The sections were deparaffinized in xylene and rehydrated through a graded ethanol series. Whole kidney coronal sections from each animal were digitally imaged at 20× magnification using the Grundium Ocus (Grundium Ltd., Finland).

#### 2.5.1 Glomerular Density

Samples were stained with periodic acid shifts reagent (PAS) using an established protocol and counterstained with Mayer's Haematoxylin. Glomerular density was quantified as the number of glomeruli per cortical area. Cortical area was segmented by a single user using Amira software (Thermo Fisher Scientific, Waltham, MA), as previously described [18, 23]. Cortex area was defined as the total kidney tissue excluding the medulla, with the corticomedullary boundary approximated by the glomerular boundary.

#### 2.5.2 Proximal Tubule Fraction

Lotus lectin staining was performed as described in our previous work to highlight the brush border of proximal tubules, with sections counterstained with methylene blue [18, 23]. For quantification, 10–12 representative images were taken at a uniform depth of 20 μm within cortex using a DM1000 LED microscope (Leica Microsystems, Germany) and DMC6200 digital camera. Proximal tubule fraction was assessed by quantifying the area occupied by “non–proximal tubule” structures in subcapsular regions using Amira software, following previously published methods (Cwiek et al., Figure S2b) [18, 23]. “Non‐proximal tubule” areas included glomeruli, other tubule sections, interstitial space, and vasculature.

#### 2.5.3 Atubular Glomeruli

Lotus‐lectin‐stained samples were also examined for disruptions in the glomerulotubular junction. Glomeruli were categorized as Lotus‐positive to indicate a functional connection between the glomerulus and the tubule, or Lotus‐negative, which serves as a surrogate biomarker for atubular glomeruli (ATGs). The percentage of ATGs was calculated by dividing the number of Lotus‐negative glomeruli by the total number of glomeruli (Cwiek et al. Figure S2d) [18, 23]. Baseline sex differences in the percentage of Lotus‐negative glomeruli have been reported [27, 28]. To account for this and allow for further comparison between sexes, ATG percentages were normalized within each sex using the T‐ND group as a reference by subtracting the median ATG percentage of the corresponding sex's T‐ND group from each individual's ATG percentage.

#### 2.5.4 Renin

Staining followed an established protocol [29]. Glomeruli with renin staining in the afferent arteriole were classified as “renin positive”. The percentage of renin‐positive glomeruli over the total number of glomeruli was used to quantify renin expressing cells.

#### 2.5.5 Podocyte Density

Dual immunofluorescence staining for WT‐1 (Invitrogen, Rabbit Anti‐WT1, 1:50 dilution) and synaptopodin (Acris Antibodies Mouse Anti‐Synaptopodin, Clone G1D4, BM5086, 1:20 dilution) was performed. Nuclei were stained with Hoescht. Podocyte density and glomerular area were assessed using a protocol adapted from Venkatareddy et al., as previously described [18, 30]. Investigators (KJR, JI), blinded to the animal's condition, randomly selected approximately 10 glomeruli per kidney for quantification to ensure representative sampling of the 2D section. Image J analytic software (National Institutes of Health) was employed to measure the apparent mean diameter of WT‐1+ podocyte nuclei. Synaptopodin staining was used to determine glomerular area. Podocyte density was calculated as described previously [30].

### 2.6 Glomerular Number and Glomerular Area

We applied cationic ferritin‐enhanced magnetic resonance imaging (CFE‐MRI) to measure N_glom_ in the kidneys of the male mice in this model, as described previously [18, 23, 31]. However, we could not measure N_glom_ in the images of kidneys of the female mice due to “CF leakage”, which obscured the glomerular boundaries and hindered the ability to distinguish individual glomeruli and accurately quantify N_glom_. Instead, N_glom_ was determined stereologically using the Weibel‐Gomez method, which has been previously validated as a suitable substitute for CFE‐MRI [18, 32].

MRI was used to measure cortical volume, as previously described [18]. Following euthanasia, kidneys were placed in a customized container for MRI. Scans were performed on a 9.4 T MRI (Bruker, Billerica, MA). A 3D gradient recalled echo (GRE) pulse sequence with a repetition time (TR) of 90 ms, echo time (TE) of 15 ms, field of view of 28 × 42 × 28 mm, matrix size of 512 × 768 × 512, flip angle 30°, and isotropic spatial resolution of 55 × 55 × 55 μm^3^ was used to acquire images. The raw images were manually segmented with Amira Software to obtain kidney and medullary volume to calculate cortical volume which was used to measure glomerular number by stereologic methods described below.

Following MRI, the imaged kidney was bisected along the sagittal plane, and both halves were embedded in paraffin wax for histological processing and stereological analysis. Cortex, medulla, and glomeruli were segmented from PAS‐stained sections (*n =* 3/kidney) using Amira Software. Each analysed section was a minimum of 100 μm apart to measure nonoverlapping glomeruli and ensure a representative sample of the kidney. Staining and imaging procedures were performed as described above. MATLAB (The Mathworks, Natick MA, https://mathworks.com) was used to perform all calculations. Glomerular number calculations were conducted using established methods with a size distribution coefficient *k* = 1.04 and shape coefficient β = 1.38 [32]. Glomerular area was quantified as the average cross‐sectional area (μm^2^) of all segmented glomeruli from the three histologic sections per kidney.

### 2.7 Bulk RNA Sequencing

Library preparation and sequencing: Whole kidney RNA sequencing (RNA‐seq) was performed on 3–4 samples per group. After the dissection, the kidneys were stored in RNAlater Stabilization Solution (Sigma Aldrich, St. Louis, MO) at −80°C until use. Total RNA was extracted from whole kidneys at 18 weeks using the TRIzol Reagent (Invitrogen, Waltham, MA), followed by cleanup with the RNeasy MinElute Cleanup Kit with RNase‐Free DNase (QIAGEN, Hilden, Germany). Total RNA was eluted in RNase‐free water (final volume 50 μL) and stored at −80°C until analysis. A NanoDrop Spectrophotometer (Thermo Fisher, Waltham, MA) was used to measure RNA concentration and OD260/280 prior to sequencing. To minimize the effect of variations in cellular composition, the whole kidney of each mouse was used for total RNA extraction. Extracted RNA samples that met the purity criteria RNA integrity number [RIN] > 4.0 according to the Agilent 2100 Bioanalyzer were subjected to un‐stranded mRNA‐Seq library preparation. The library preparation and sequencing were performed by specialists at Novogene Inc. (Sacramento, CA). Reverse transcription and library preparation were performed using the NEBNext Ultra II RNA Library Prep Kit for Illumina. Sequencing flow‐cell was carried out using the Illumina NovaSeq 6000 S4 Flowcell. The minimum RIN value used reflects the standard general threshold defined by Novogene for eukaryotic RNA sequencing. RIN values for the obtained samples ranged from 5.9–8.2 (Table S2). Additional RNA quality control metrics are reported in Table S2 and Figure S1. The data discussed in this publication have been deposited in NCBI's Gene Expression Omnibus and are accessible through GEO Series accession number GSE319377 (www.ncbi.nlm.nih.gov/geo/query/acc.cgi?acc=GSE319377).

#### 2.7.1 Data Analysis

After removing low‐quality sequencing reads that failed the quality check and after trimming adapter sequences, the obtained sequences were aligned to the mouse GRCm39 reference genome and quantified gene expression by transcript counting (GENCODE release M36) using the STAR aligner [33]. Differential expression was determined using the Bioconductor package DESeq2 [34]. We eliminated genes with total read counts of 10 or fewer across all samples from the analysis. Significantly differentially expressed genes (DEGs) were determined based on a log_2_‐fold change of less than −1 or greater than 1 and a false discovery rate (FDR) adjusted *p*‐value less than 0.05. DEGs were further analysed for their biological functions through Gene Ontology (GO) enrichment analysis using a Bioconductor package, clusterProfiler [35, 36]. Biological Process (BP) and molecular function (MF) GO terms were used to assess enrichment. GO term over‐representations were assessed using a hypergeometric distribution for GO enrichment, and a permutation test (eps = 1e‐10) for the gene set enrichment (GSE). A GO term with a *q*‐value of less than 0.05 was considered significantly enriched.

### 2.8 Quantitative Real‐Time Polymerase Chain Reaction

First‐strand complementary DNA was synthesized using the High‐Capacity cDNA Reverse Transcription Kit (Thermo Fisher Scientific, Waltham, MA) according to the manufacturer's instructions. Quantitative real‐time polymerase chain reaction (qRT‐PCR) was performed in duplicate using the CFX Opus 384 Real‐Time PCR System with iTaq Universal SYBR Green Supermix (BIO‐RAD). Primer sets were designed using the Primer3Plus (https://www.primer3plus.com/). Gene expression levels were normalized to the housekeeping gene *Gapdh*. Primer sequences are listed in Table S3.

### 2.9 Sex Differences Analysis

The experimental design and data from the male cohort have been published [18]: term nondiabetic (T‐ND; *n* = 7), preterm nondiabetic (PT‐ND; *n* = 6), term diabetic (T‐D; *n* = 5), and preterm diabetic (PT‐D; *n* = 6).

### 2.10 Statistical Analysis

Prism 10.4 software (GraphPad) was used to analyse differences across experimental groups. All statistical tests for structural and functional analyses in Prism were conducted using two‐tailed Mann–Whitney *U* tests, except for urine ACR analysis, which used a one‐tailed Mann–Whitney *U* test because albuminuria is common in DKD. Statistical tests for the results of qRT‐PCR were conducted using one‐tailed Mann–Whitney *U* tests in Prism to validate upregulated DEGs in PT‐D females vs. T‐D females. Sex differences within individual experimental groups (T‐ND, PT‐ND, T‐D, and PT‐D) were also analysed in Prism. Further analysis of sex‐specific effects was conducted in R (Version 4.4.1). Two comparisons were made: (1) PT‐D vs. T‐ND to evaluate the combined effect of preterm birth and diabetes, and (2) PT‐D vs. T‐D to assess the effect of preterm birth in the context of diabetes. Additional analyses of PT‐ND vs. PT‐D and T‐ND vs. T‐D were performed and included in Supporting Information. For both comparisons, the magnitude of differences between groups was compared between males and females to identify sex‐specific responses. *p*‐values < 0.05 were considered significant.

## 3 Results

### 3.1 Model Characterization

At 15 weeks of age, diabetic mice began to lose weight (Figure S2a), consistent with the male cohort [18] and other STZ‐induction models [24]. The PT‐D and T‐D body weights (BW) were lower than PT‐ND and T‐ND, respectively (Figure S2b). Following STZ treatment, T‐D and PT‐D mice both had a higher terminal blood glucose than T‐ND and PT‐ND, respectively (Figure S2c). All T‐D mice developed hyperglycemia (blood glucose > 300 mg/dL) following the initial STZ treatment. Three PT‐D mice required a second round of STZ, as recommended by the Animal Models of Diabetic Complications Consortium protocol if the blood glucose concentration was < 300 mg/dL at 6 weeks after the first STZ injection [20]. Mice that required a second round of STZ were included in the analysis because there was no difference in average blood glucose observed between the T‐D (441 ± 74 mg/dL) and PT‐D (377 ± 184 mg/dL) mice at 18 weeks (Figure S2d).

### 3.2 Effects of Preterm Birth and Diabetes in Female Mice

We compared the preterm with diabetes (PT‐D) group to the term without diabetes (T‐ND) group to assess the combined effects of preterm birth and diabetes. PT‐D had a greater kidney weight to body weight (KW/BW) than T‐ND with no difference in absolute kidney weight (KW) (Figure S2e,f). The urine ACR was higher in PT‐D than T‐ND (Figure S2h) with no difference in GFR (Figure S2g) or blood urea nitrogen (BUN) concentration (Figure S2i).

### 3.3 Structural Alterations: PT‐D vs. T‐ND

**FIGURE 2:**
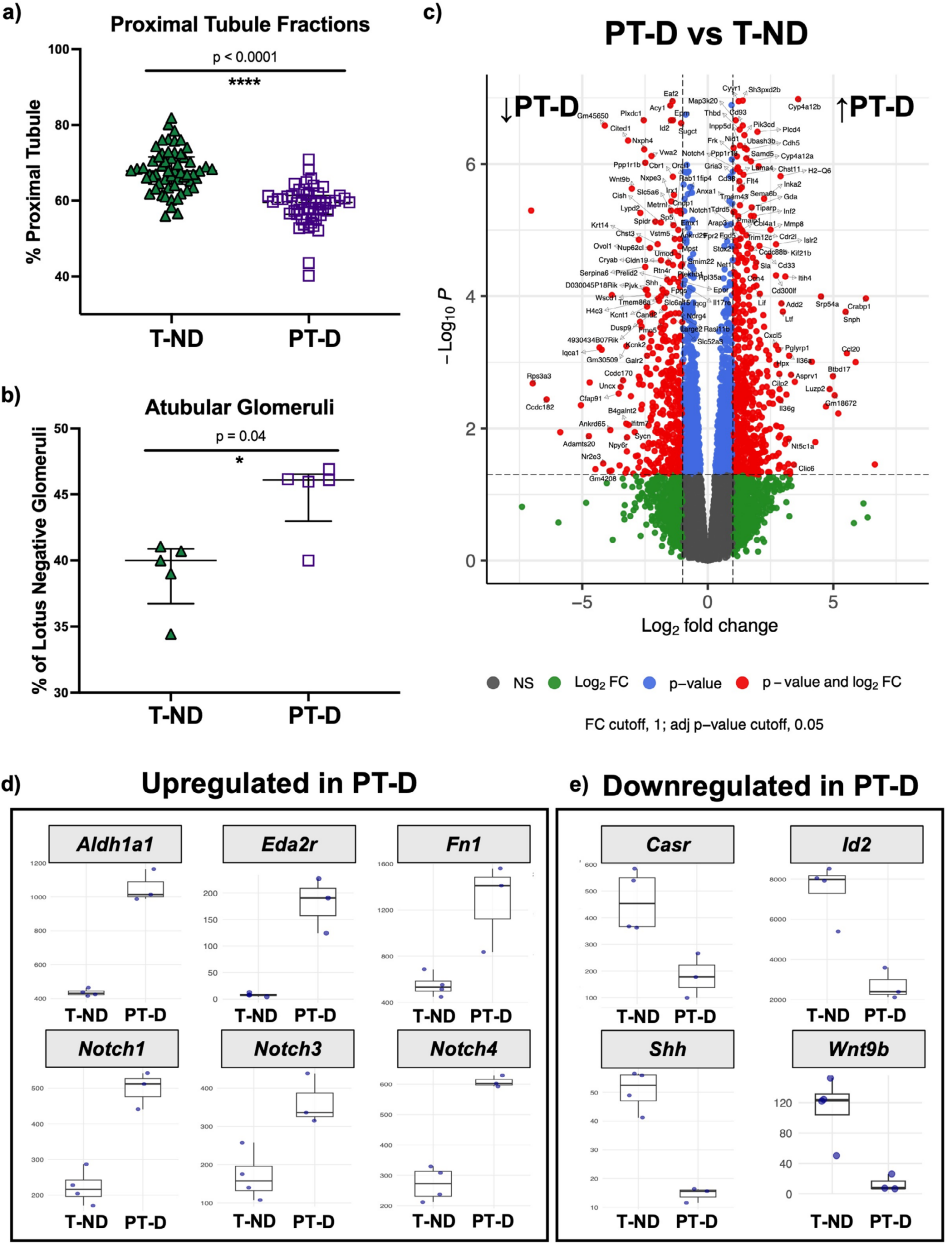
Structural and transcriptional alterations in the kidney following preterm birth and exposure to STZ‐induced hyperglycemia in female mice. (a) Proximal tubule fraction was lower in preterm diabetic (PT‐D) than in term non‐diabetic (T‐ND) females. (b) The percentage of atubular glomeruli was greater in PT‐D than T‐ND. (c) Differentially expressed genes (DEGs) between PT‐D and T‐ND animals reveal differences in gene expression. (d) Genes related to inflammation, fibrosis, apoptosis, and Notch signaling pathway were upregulated in PT‐D compared to T‐ND. (e) Genes involved in developmental processes, anti‐inflammatory responses, and anti‐fibrotic pathways were downregulated in PT‐D compared to T‐ND. Enriched pathways and related genes are fully depicted in Gene Ontology (GO) analysis in Figure 3. Two‐tailed Mann–Whitney tests (a, b) with a *p*‐value < 0.05 considered statistically significant. Fold change threshold of ±1.0 and an adjusted *p*‐value of 0.05 were used to define differentially expressed genes (c, d, e).

We performed the same histologic assessment of the female mice as the male mice reported in Cwiek et al. [18]. Proximal tubular injury occurs early in DKD [37], and animal models have shown that early kidney injury can lead to disruptions in the connection between the proximal tubule and the glomerulus [18, 27, 38, 39]. The PT‐D females had a lower fraction of proximal tubules compared to T‐ND females (PT‐D: 57.9% ± 0.6% vs. T‐ND: 68.6% ± 4.6%; Figure 2a). PT‐D females also had a higher proportion of atubular glomeruli (ATGs) compared to T‐ND females (T‐ND: 40.0% ± 1.7%; PT‐D: 46.1% ± 0.2%, Figure 2b). There was no difference in glomerular density (Figure S2j), percentage of renin positive glomeruli (Figure S3a), podocyte density (Figure S3b), or average glomerular area (Figure S3d). There was no difference in glomerular number (N_glom_) between groups (PT‐D: 13,911 (11,177‐15,046) vs. T‐ND: 11,502 (10,337‐11,610)) (Figure S3c).

### 3.4 Transcriptome Alterations: PT‐D vs. T‐ND

A total of 968 genes were differentially expressed in PT‐D compared to T‐ND, including 577 upregulated and 391 downregulated genes (Figure 2c–e). The complete list of DEGs is provided in Table S4.

**FIGURE 3:**
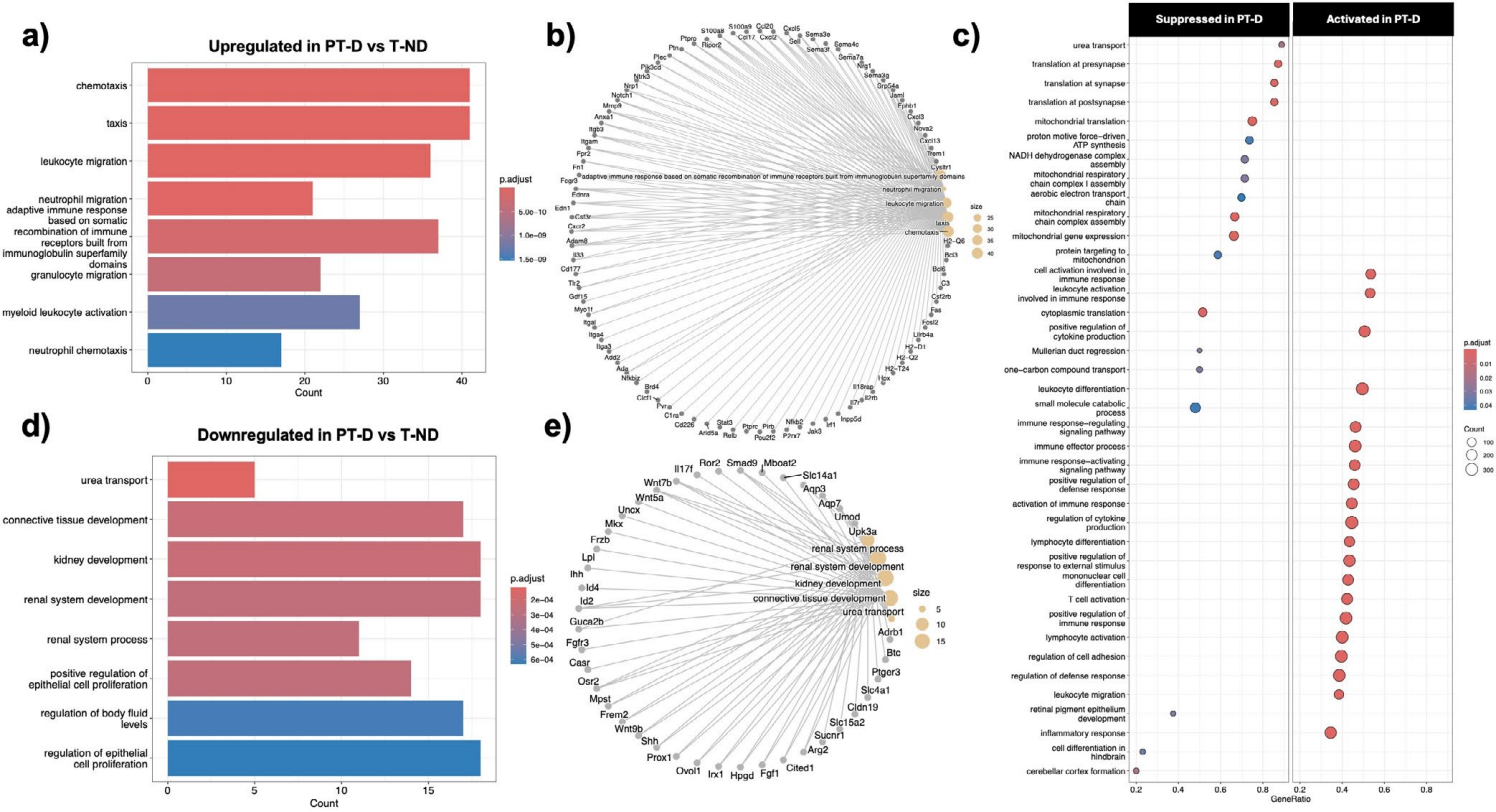
Gene Ontology analyses of Bulk RNA‐Seq for differentially expressed genes between T‐ND and PT‐D: Biological Process GO terms. (a) GO enrichment analysis showed that immune‐related GO terms were enriched in PT‐D upregulated genes, and (d) developmental‐related GO terms were enriched in PT‐D downregulated genes. (b, e) The linkages of corresponding genes and those terms are shown in the CNET plots. (c) Gene Set Enrichment analysis revealed suppression of mitochondrial‐associated pathways and activation of immune‐ and inflammation‐related pathways in the PT‐D group compared to T‐ND.

Gene ontology (GO) enrichment analysis was performed to identify processes and functions altered by the dual exposure of preterm birth and hyperglycemia. The enriched Biological Processes GO terms are shown in Figure 3. The enriched Molecular Function GO terms are shown in Figure S4. Immune‐related GO terms were enriched in upregulated genes in the PT‐D group compared to T‐ND, including “leukocyte migration,” “neutrophil migration,” and “adaptive immune response based on somatic recombination of immune receptors built from immunoglobulin superfamily domains” (Figure 3a,b). Gene set enrichment analysis revealed activation of several pathways in PT‐D compared to T‐ND, including immune‐ and inflammation‐related pathways and suppression of several mitochondria‐associated pathways (Figure 3c).

Downregulated genes in PT‐D compared to T‐ND were enriched for GO terms related to developmental biological processes, including “kidney development”, “renal system development”, “renal system processes”, and “connective tissue development” (Figure 3d,e). Key kidney development genes with reduced expression in PT‐D included*: Cited1*, *Fgf1*, *Prox1*, *Shh*, and *Wnt9b* (Figure 2e). The expression of anti‐inflammatory and anti‐fibrotic genes (*Casr*, *Id2*, and *Lpl* (Figure 2e)) was reduced in PT‐D compared to T‐ND, suggesting a reduction in the protective pathways in the preterm diabetic kidney [40, 41, 42].

### 3.5 Effects of Preterm Birth in the Setting of Diabetes in Female Mice

To isolate effects attributable to preterm birth, the preterm‐diabetics (PT‐D) were compared to term‐diabetics (T‐D). There were no differences between groups for KW, KW/BW, GFR, or urine ACR (Figure S2e–h). However, BUN was higher in PT‐D compared to T‐D (Figure S2i).

### 3.6 Structural Alterations: PT‐D vs. T‐D

**FIGURE 4:**
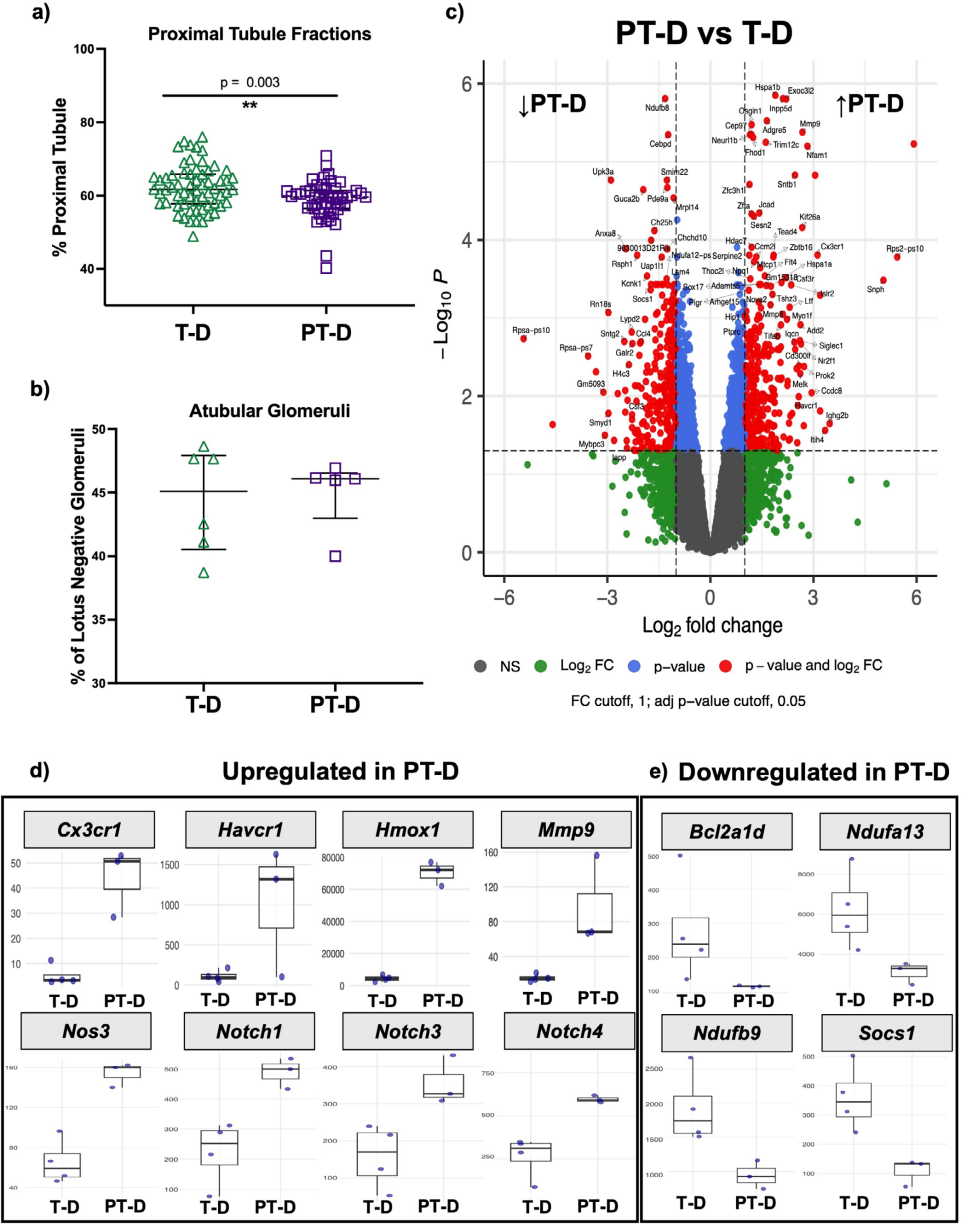
Structural and transcriptional alterations in the kidney following preterm birth in mice with hyperglycemia. (a) Proximal tubule fraction was lower in preterm diabetic than term diabetic females. (b) There was no difference in the percentage of atubular glomeruli between preterm diabetic and term diabetic females. (c) Differentially expressed genes (DEGs) between preterm diabetic and term diabetic animals reveal differences in gene expression. (d) Genes related to angiogenesis, inflammation, fibrosis, and Notch signaling pathway were upregulated in PT‐D compared to T‐D. (e) Genes involved in mitochondrial activity, cellular respiration, and anti‐fibrotic pathways were downregulated in PT‐D compared to T‐D. Enriched pathways and related genes are fully depicted in Gene Ontology (GO) analysis in Figure 5. Two‐tailed Mann–Whitney tests (a, b) with a *p*‐value < 0.05 considered statistically significant. Fold change threshold of ±1.0 and an adjusted *p*‐value of 0.05 were used to define differentially expressed genes (c, d, e).

PT‐D females had lower proximal tubule fractions compared to T‐D females (PT‐D: 57.9% ± 0.6%, T‐D: 62.6% ± 4.2%; Figure 4a), but the proportion of ATGs was not different (PT‐D: 46.1% ± 0.2%, T‐D: 45.1% ± 6.2%; Figure 4b). Glomerular density (Figure S2j), the percentage of renin positive glomeruli, podocyte density, N_glom_, and glomerular area were not different (Figure S3a–d).

### 3.7 Transcriptome Alterations: PT‐D vs. T‐D

Transcriptional comparisons of PT‐D and T‐D females revealed 668 DEGs, including 346 upregulated genes and 322 downregulated genes (Figure 4c–e). The complete list of DEGs is shown in Table S5. To validate these results, qRT‐PCR was performed on three genes (Figure S5).

**FIGURE 5:**
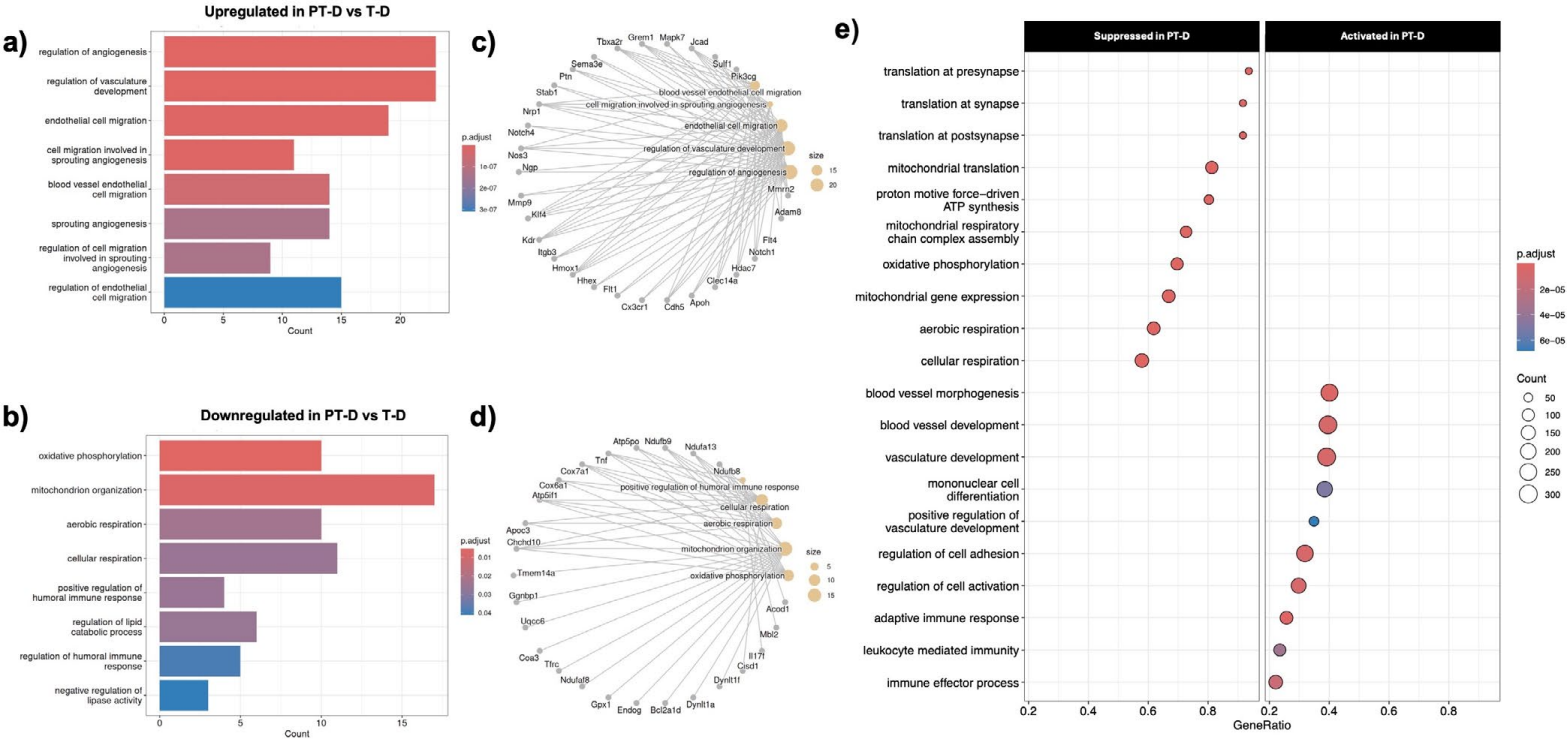
Gene Ontology analyses of Bulk RNA‐Seq for differentially expressed genes between T‐D and PT‐D: Biological Process GO terms. (a) GO enrichment analysis revealed enrichment of the GO terms “regulation of angiogenesis” and “regulation of vascular development” in genes upregulated in PT‐D compared to T‐D. (b) GO terms associated with mitochondrial activity and cellular respiration were enriched in downregulated PT‐D genes relative to T‐D. (c, d) The linkages of corresponding genes and those terms are shown in the CNET plots. (e) Gene Set Enrichment analysis revealed activation of angiogenesis, vascular development, and immune‐related pathways and suppression of mitochondrial‐associated pathways in the PT‐D group compared to T‐D.

GO enrichment analysis was performed to identify processes and functions altered by preterm birth in the setting of hyperglycemic exposure. Enriched biological processes GO terms are shown in Figure 5, and enriched molecular function GO terms are shown in Figure S6. Pathway analysis revealed dysregulation of angiogenesis and vascular development in PT‐D compared to T‐D, as evidenced by enrichment of GO terms such as “regulation of angiogenesis”, “regulation of vascular development”, and “endothelial cell migration” in upregulated PT‐D genes compared to T‐D. Angiogenesis plays a critical role in both diabetes and kidney development. Diabetes is known to impair angiogenic signalling and vascular integrity [43], and proper vascular development is globally impaired by preterm birth, with a particular effect on the kidneys [44, 45]. Consistent with these findings, several angiogenesis‐related genes were upregulated in PT‐D, including *Cdh5*, *Flt1*, *Flt4*, *Hmox1*, *Nos3*, and *Sema3e* (Figure 5c). Results from qRT‐PCR showed that *Hmox1* and *Nos3* were expressed more in the kidneys of PT‐D females compared to T‐D, validating these findings (Figure S5a,b). Gene set enrichment analysis also indicated activation of pathways in PT‐D related to angiogenesis, vasculature, and blood vessel development compared to T‐D. *Hmox1* was markedly upregulated in PT‐D compared to T‐D, showing both the most significant adjusted *p*‐value and a large fold change. *Hmox1*, which encodes for heme oxygenase‐1 (HO‐1), is involved with angiogenesis [46]. *Hmox1* is induced by hypoxia‐inducible factor‐1 (HIF‐1), and HO‐1 overexpression enhances VEGF synthesis [47, 48, 49].

In PT‐D kidneys, several GO terms associated with mitochondrial activity and cellular respiration, such as “mitochondrion organization,” “cellular respiration,” and “aerobic respiration”, were enriched in downregulated genes in the T‐D group (Figure 5b,d). Downregulated genes within these GO terms included *Apoc3*, *Bcl2a1d*, *Gpx1, Ndufa13, and Ndufb9* [50, 51, 52]. Altered metabolism is known to contribute to DKD progression. Collectively, these data, including the lower proximal tubular fraction and upregulation of genes associated with DKD progression and damage, support that female PT‐D have more injury with hyperglycemia compared to female T‐D mice.

*The DEGs for* secondary analyses of T‐ND vs. T‐D are included in Supplementary Table 6 and Figure S7. *The DEGs for* secondary analyses of PT‐ND vs. PT‐D are included in Table S7 and Figure S7.

### 3.8 Sex Differences

**FIGURE 6:**
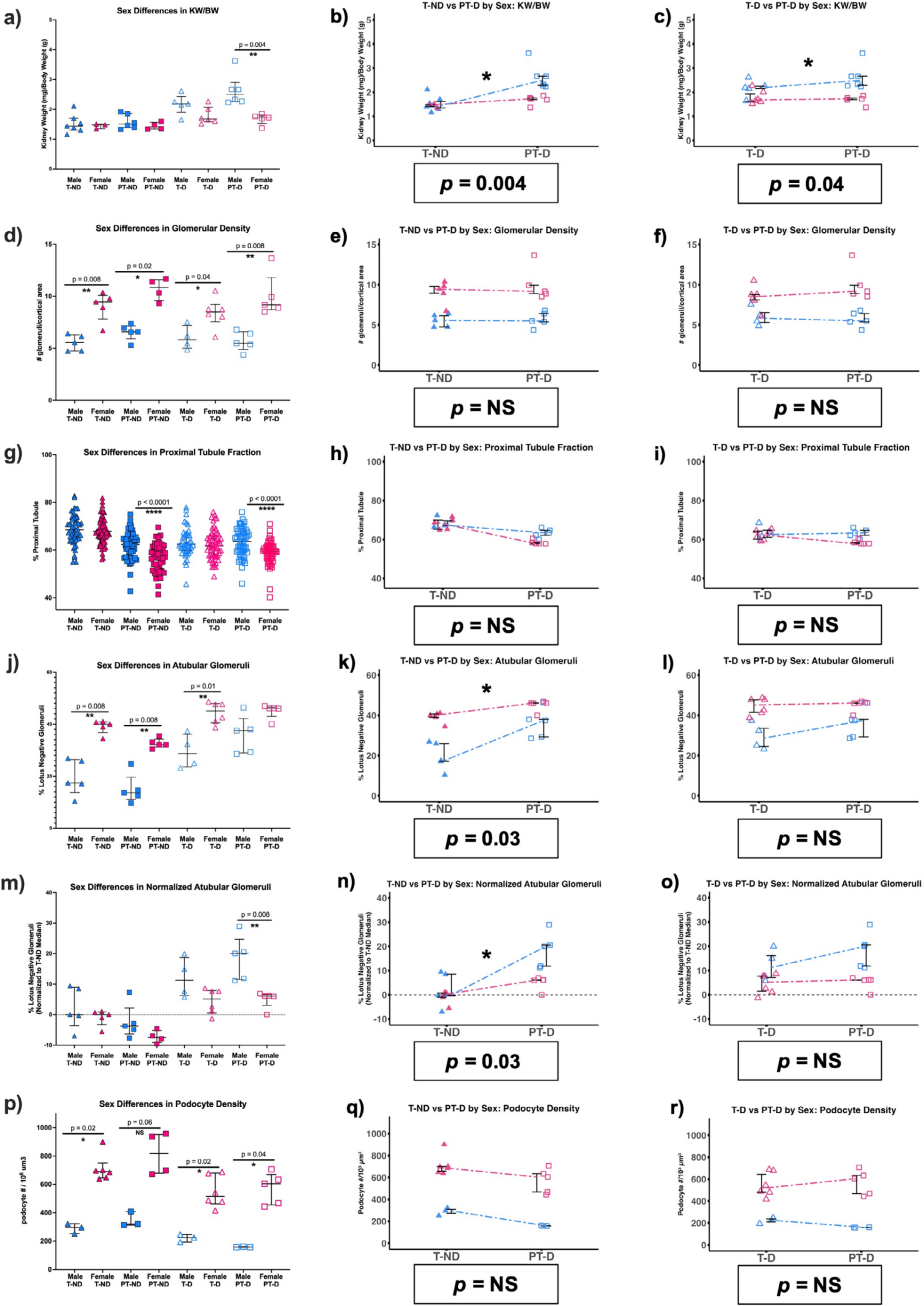
Characterization of structural sex differences in the model. (a) Preterm diabetic males had a greater kidney weight to body weight ratio (KW/BW) than preterm diabetic females. (b) A sex difference in KW/BW was observed between the term non‐diabetic and preterm diabetic groups, with male animals exhibiting a greater increase than female animals. (c) A sex difference in KW/BW was also observed between the term diabetic and preterm diabetic groups, with male animals exhibiting a greater increase than female animals. (d) Female animals across all conditions had a greater glomerular density than males. (e) No sex‐specific differences in glomerular density were observed between term non‐diabetic and preterm diabetic animals, or (f) between term diabetic and preterm diabetic animals. (g) Female preterm animals, with and without diabetes, had a lower proximal tubule fraction than their respective male comparisons. (h) No sex‐specific differences in proximal tubule fraction were observed between term non‐diabetic and preterm diabetic animals, or (i) between term diabetic and preterm diabetic animals. (j) Female animals had a greater baseline percentage of lotus negative glomeruli, as evidenced by the higher percentage of atubular glomeruli in term non‐diabetic females compared to term non‐diabetic males. There was no sex difference in atubular glomeruli between preterm diabetic animals, without normalizing for the baseline difference in lotus negative glomeruli. (k) A sex difference in atubular glomeruli (without normalization) was observed between the term non‐diabetic and preterm diabetic groups, with male animals exhibiting a greater increase than female animals. (l) No sex‐specific differences in atubular glomeruli were observed between term diabetic and preterm diabetic animals. (m) Following normalization to the median atubular glomerular percentage in term non‐diabetic animals in each sex, preterm diabetic males had a greater percentage of atubular glomeruli than preterm diabetic females. (n) The sex‐specific difference in atubular glomeruli between term non‐diabetic and preterm diabetic animals was still observed after normalization. (o) Normalization for atubular glomeruli did not influence the absence of sex differences between term diabetic and preterm diabetic animals. (p) Podocyte density was greater in female animals across all conditions, although the difference between preterm non‐diabetic animals did not reach significance. (q) No sex‐specific differences in podocyte density were observed between term non‐diabetic and preterm diabetic animals, or (r) between term diabetic and preterm diabetic animals. Two‐tailed Mann–Whitney tests (a, d, g, j, m, p) with a *p*‐value < 0.05 considered statistically significant. The difference in contrasts (1) T‐ND vs. PT‐D (b, e, h, k, n, q) and (2) T‐D vs. PT‐D (c, f, I, l, o, r) were assessed for sex differences with a *p*‐value 0.05 considered statistically significant.

Sex differences in injury markers and histopathological features of DKD have been reported in other animal models [53]. At baseline, there was a significant difference between the male T‐ND and female T‐ND in glomerular density, ATG's and podocyte density. The observed sex‐specific differences are presented below, with a focus on PT‐D males and PT‐D females (Figure 6; Figure S8). We also examined the effect of preterm birth in diabetic mice based on sex by comparing the magnitude of the difference between PT‐D and T‐D mice in males versus females (Figure 6).

### 3.9 Structural Alterations: Sex Differences

There was no difference in KW/BW between the sexes in all the ND mice. However, PT‐D males had a greater KW/BW than PT‐D females (Figure 6a). Both male and female PT‐D groups had a greater KW/BW compared to T‐ND for their respective sex. These sex‐specific differences in KW/BW between PT‐D and T‐ND and PT‐D and T‐D were statistically significant (Figure 6b,c).

Female mice had a significantly higher glomerular density than males across all groups (T‐ND males: 5.56 ± 1.41; T‐ND females: 9.45 ± 0.86; PT‐ND males: 6.58 ± 0.39; PT‐ND females: 10.86 ± 1.38; T‐D males: 5.82 ± 1.23; T‐D females: 8.50 ± 0.66; PT‐ND males: 5.48 ± 1.03; PT‐ND females: 9.17 ± 1.03 glomeruli/μm^2^) (Figure 6d). There was no significant difference in glomerular density between PT‐D and T‐ND or PT‐D and T‐D in either sex (Figure 6e,f), indicating that higher glomerular density is a baseline sex difference.

There was no difference in proximal tubule fraction between the males and female T‐ND mice; however, female PT‐ND had a lower proximal tubule fraction than PT‐ND males (Figure 6g). In both sexes, the dual exposure to preterm birth and hyperglycemia resulted in a lower proximal tubule fraction compared to T‐ND of the same sex (Figure 6h). Among males, PT‐D and T‐D had a similar proximal tubule fraction, whereas PT‐D females had a lower proximal tubule fraction compared to T‐D females (Figure 6h). The difference between T‐D and PT‐D mice was not statistically different between the sexes, indicating that the lower proximal tubule fraction seen in the PT‐D females compared to T‐D females was not attributable to sex (Figure 6i).

Consistent with prior reports that males have a higher fraction of lotus positive cells in Bowman's capsule than females (34), the baseline proportion of ATGs was different between sexes; T‐ND females had a greater proportion of ATGs than T‐ND males (Figure 6j). To account for baseline sex differences, ATG proportions were normalized within each sex using the median value from T‐ND mice of the corresponding sex. After normalization, PT‐D females had a smaller proportion of ATGs than PT‐D males (Figure 6m). In both sexes, there was a higher proportion of ATG in PT‐D mice compared to T‐ND, but the difference was significantly greater in males than females (Figure 6k,n). Although PT‐D males had a greater proportion of ATGs than PT‐D females, the difference between PT‐D and T‐D was not statistically different between sexes (Figure 6l,o).

Female mice had a greater podocyte density than males across all conditions, although the difference between male and female PT‐ND mice did not reach statistical significance (Figure 6p). This sex difference in podocyte density has also been reported in human studies [54]. Podocyte density was not significantly different between PT‐D and T‐ND females, whereas PT‐D males had a lower podocyte density than T‐ND males. Similarly, podocyte density was similar between PT‐D and T‐D females, whereas PT‐D males had a lower podocyte density than T‐D males. Despite apparent sex differences, the magnitude of the difference between PT‐D and T‐ND, as well as between PT‐D and T‐D mice, was not significantly different between sexes (Figure 6q,r).

### 3.10 Transcriptome Alterations: Sex Differences

**FIGURE 7:**
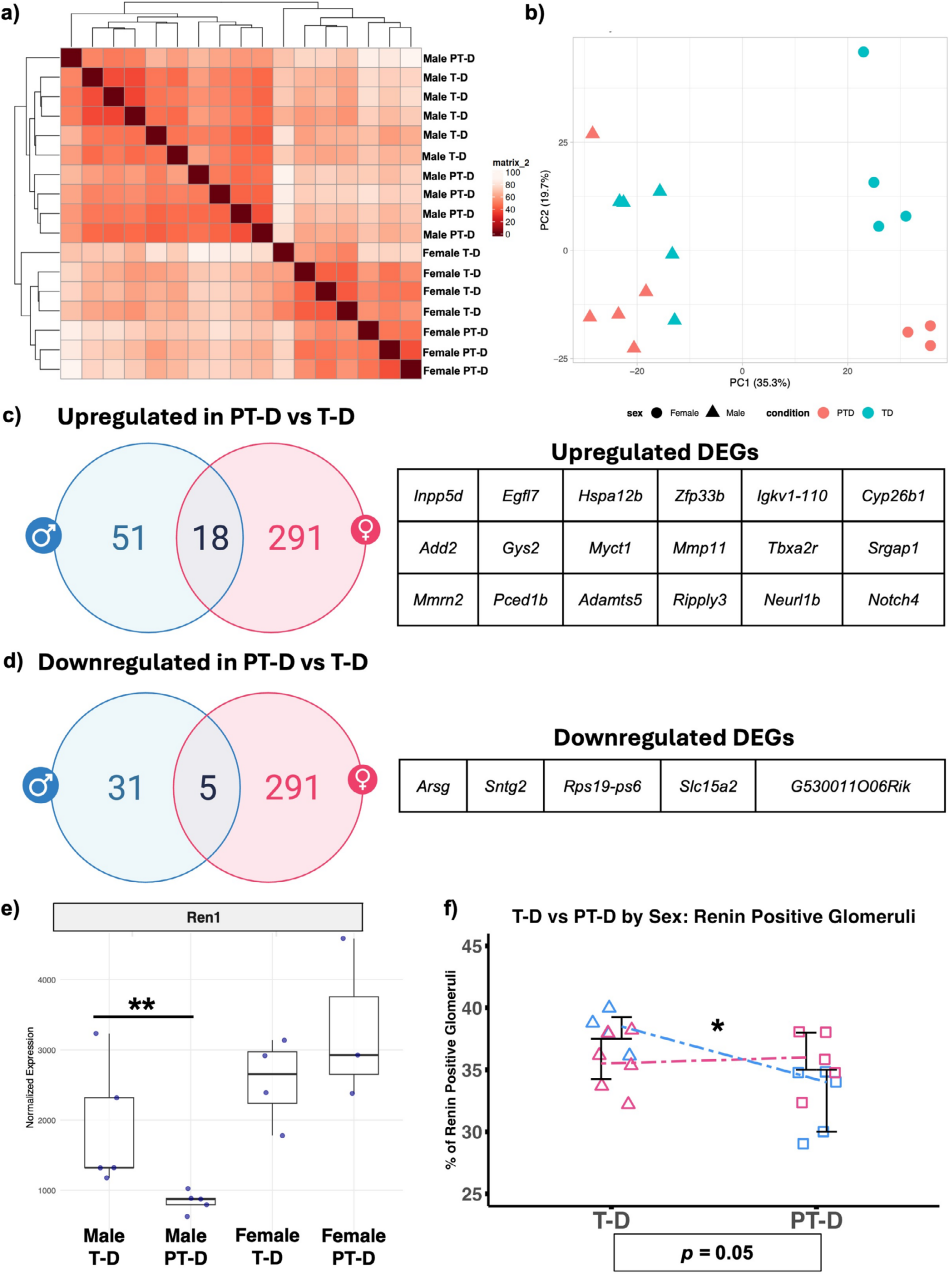
Dual exposure to preterm birth and hyperglycemia reveals sex‐specific transcriptomic alterations. (a) Hierarchical clustering analysis of Bulk RNA‐Seq data reveals the expression profiles clustering into four groups. (b) A principal component analysis also showed clear sexual dimorphism. (c, d) Twenty‐three differentially expressed genes were shared across sexes in the PT‐D groups when compared to T‐D. Both male and female PT‐D mice showed Notch signalling abnormalities. (e) There was less *Ren1* expression in preterm diabetic males compared to term diabetic males. There was no significant difference between females. (f) This sex difference in renin expression was validated by IHC.

We performed RNA‐seq analysis on the male and female kidneys to identify molecular changes in the kidney attributable to preterm birth in the setting of hyperglycemia. The expression profiles clustered into four groups, except one sample, by hierarchical clustering (Figure 7a). Principal component analysis (PCA) of diabetic mice (T‐D and PT‐D) revealed a clear distinction between male and female samples, with PC1 accounting for 35.3% of the total variance. PC2, accounting for 19.7% of the variability, was correlated with sample condition. Females exhibited a more pronounced condition‐dependent segregation along PC2 compared to males (Figure 7b). Sex‐stratified PCA including T‐ND, PT‐ND, T‐D, and PT‐D is available in Figure S9.

We identified 605 DEGs between female PT‐D and T‐D kidneys. Of those, 291 were identified as female‐specific upregulated genes and 291 female‐specific downregulated genes in PT‐D compared to T‐D. Eighteen were consistently upregulated in both male and female PT‐D compared to T‐D (Figure 7c), while five DEGs were consistently downregulated in both sexes (Figure 7d). Bulk RNA‐Seq identified alterations in the Notch signalling across both sexes, evidenced by enrichment of the GO terms “Notch signaling pathway” and “negative regulation of Notch signaling pathway” in PT‐D compared to T‐D (Figure S10). While baseline expression of *Notch1*, *Notch3*, and *Notch4* was similar between T‐D males and females, all three genes were significantly upregulated in PT‐D females. The increased *Notch1* expression in PT‐D females as compared to T‐D females was validated by qRT‐PCR (Figure S5c). Additionally, *Notch4* was significantly upregulated in PT‐D males, with *Notch1* and *Notch3* showing non‐significant trends toward upregulation. We previously reported that *Ren1*, which encodes renin, was downregulated in PT‐D males compared to T‐D males with validation by IHC staining [18]. However, in the females, there was no difference in *Ren1* expression or protein levels (Figure 7e,f).

## 4 Discussion

**FIGURE 8:**
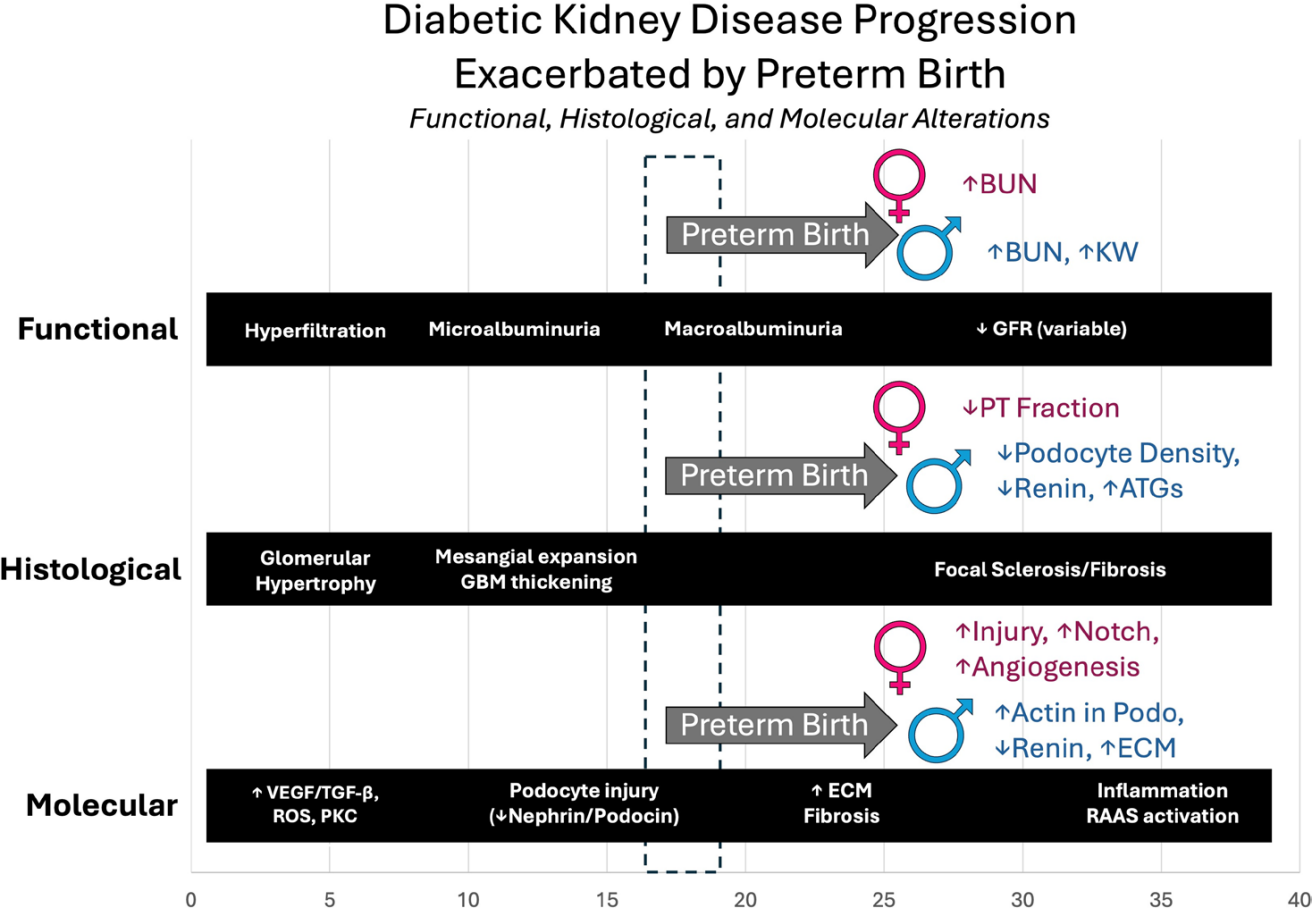
Diabetic kidney disease progression is exacerbated by preterm birth. Preterm birth exacerbated functional, histologic, and molecular alterations in the kidneys of diabetic mice. Alterations associated with the progression of diabetic kidney disease (DKD) in CD‐1 mice exposed to STZ are represented by the black bars. Kidney alterations are assessed in mice euthanized at 18 weeks of age, after a 12‐week exposure to hyperglycemia induced by STZ at 6 weeks. The timeframe of this study is indicated by the grey dashed box. In our model, preterm diabetic female mice had less severe alterations than preterm diabetic male mice.

In this dual exposure model of preterm birth and hyperglycemia, female mice exhibited mild alterations in kidney structure and function, with early molecular changes consistent with diabetic kidney injury. The combination of preterm birth and hyperglycemia resulted in a higher BUN and reduced proximal tubule fraction compared to term females exposed to hyperglycemia. Transcriptomic analysis revealed upregulation of angiogenesis pathways and the Notch pathway along with downregulation of mitochondrial function in preterm diabetic compared to term diabetic females, consistent with a more advanced DKD phenotype in those born preterm. These data also confirm several sex differences at baseline and reveal a greater increase in normalized kidney weight in the preterm males with diabetes, despite more atubular glomeruli, compared to preterm females with diabetes. Our previous study demonstrated that preterm males with diabetes compared to term males with diabetes had a lower podocyte density, higher BUN, and evidence of extracellular matrix gene expression changes, evidence of early DKD which was absent in the preterm females with diabetes. This study provides evidence that preterm birth increases susceptibility to hyperglycemia‐induced kidney injury in female mice, but the impact in the females is milder than in males (Figure 8). Preterm birth appears to exacerbate kidney injury by increasing susceptibility to proximal tubule damage and disrupting angiogenesis in both sexes, while more prominently affecting podocytes and renin expression in males and increased Notch signaling in females. These sex‐specific responses highlight the importance of understanding divergent mechanisms of injury, which may inform targeted strategies to reduce the burden of progressive DKD.

Data suggest that individuals born preterm have a propensity for hyperglycemia at an early age, highlighting the importance of studying this dual‐hit model prior to the onset of overt nephropathy. The link between preterm birth and development of diabetes during adulthood has been established at a population level [3, 12]. Early strategies are necessary to prevent progression to permanent, irreversible kidney damage; however, most studies of diabetic kidney disease have not accounted for preterm birth as a contributing risk factor in disease progression. Several studies have demonstrated alterations in insulin sensitivity during childhood in those born preterm [55, 56]. In a study published in the New England Journal of Medicine, children 4–10 years old who were born preterm (*n* = 50) were found to have lower insulin sensitivity and higher acute insulin release compared to term controls (*n* = 22) [55]. Here, we modelled these early manifestations of insulin insensitivity, with a short duration of hyperglycemia and mild prematurity to investigate early kidney alterations, prior to the development of significant DKD.

As expected, the dual hit of preterm birth and diabetes in the females resulted in several significant transcriptomic alterations associated with early DKD, indicating molecular changes that precede detectable structural or functional damage. Proinflammatory, profibrotic, and apoptotic genes were upregulated in preterm females with diabetes compared to term nondiabetic females, including *Col1a1* and *Fn1*. We observed alterations in pathways linked to increased oxidative stress, which plays a central role in the initiation and progression of DKD by driving renal fibrosis [57]. Notably, *Eda2r*, which was increased in the PT‐D compared to the T‐ND females, has been associated with increased oxidative stress and apoptosis in podocytes in the setting of hyperglycemia [58].

To isolate the effect of preterm birth in female mice, the PT‐D group was compared to the T‐D group. The preterm female diabetic group exhibited features consistent with a more advanced stage of DKD and a greater effect on both the endothelia and mitochondria than term diabetic mice, indicating that preterm birth in females intensifies disease progression even under similar hyperglycemic conditions. Mitochondrial dysfunction occurs early in DKD, which is particularly harmful in the proximal tubules due to their high mitochondrial content and metabolic demand [59]. Several genes linked to tubular injury, fibrosis, and inflammation were significantly elevated in PT‐D compared to T‐D, with upregulation of tubular injury genes including *Havcr1* and upregulation of profibrotic genes included: *Cx3cr1*, *Grem1*, and *Trem1*. *Melk*, associated with the regulation of TGF‐β‐mediated signalling pathways and apoptosis [60], was also increased in the PT‐D compared to T‐D females. Additionally, *Mmp9*, a mediator of extracellular matrix accumulation, podocyte injury, and albuminuria [61], was upregulated in the preterm diabetic females.

Genes important in vascular development and angiogenesis, including *Cdh5*, *Flt1*, *Flt4*, *Hmox1*, *Nos3*, and *Sema3e*, were all upregulated in preterm diabetic compared to term diabetic females. DKD progression is linked to loss of peritubular capillary rarefaction, and thus this may be indicative of greater injury in the preterm diabetic kidneys. In addition, preterm birth affects vascular development. Our previous studies using this model have demonstrated that preterm mice delivered 2 days before full term have fewer endothelial cells and have disrupted angiogenic pathways early in life [21]. This vascular phenotype has also been observed in other models, including the baboon model of preterm birth [62]. Vascular anomalies in preterm neonates also disrupt renal angiogenesis, limit perfusion of the nephrogenic zone, and increase vulnerability to hypoxia and oxidative stress [62, 63]. The effect of preterm birth on the developing vasculature can be detected in preterm neonates across their lifespan [64, 65]. Early on, preterm infants exhibit structural and functional vascular abnormalities, including impaired endothelial function, altered vascular reactivity, and increased arterial stiffness [66, 67]. For example, abnormalities in retinal vascularization underlie the development of retinopathy of prematurity [68], while disrupted pulmonary vascular development contributes to pulmonary hypertension and bronchopulmonary dysplasia [69]. In later life, systemic vascular changes, such as impaired flow‐mediated dilation and increased carotid intima–media thickness [70], have been reported in preterm survivors, suggesting a predisposition to later cardiovascular disease.

Multiple mitochondrial pathways were downregulated in preterm diabetic compared to term diabetic females, highlighting a potentially important connection between oxidative stress, mitochondrial dysfunction, and proximal tubule structural changes. Several downregulated genes and gene families in the kidneys of preterm mice with diabetes are implicated in DKD and in both acute kidney injury (AKI) and chronic kidney disease (CKD). For example, BCL‐2 regulates podocyte survival in DKD; overexpression of *Bcl‐2* in hyperglycemic cell culture reduces apoptosis, whereas decreased BCL‐2 expression has been observed in kidney biopsies from patients with DKD [71]. Similarly, genes associated with complex I subunits, including members such as *Ndufa*, *Ndufb*, and *Ndufs*, were reduced in the kidneys of preterm mice with diabetes. Impaired Complex I function is a consistent feature of DKD, where diminished activity contributes to excess ROS generation, oxidative stress, and tubular injury [72, 73]. Downregulation of Complex I proteins and *Nduf* subunits have also been seen in AKI prior to necrosis and oxidative damage, and their loss correlates with fibrosis in CKD models [74, 75, 76]. The reduction in these protective pathways, along with a less proximal tubular mass without albuminuria, may shed light on patients who develop DKD without progressive albuminuria [77].

Increased Notch expression was prominent in the preterm female diabetic mice. Several Notch receptors including *Notch1*, *Notch3*, and *Notch4* were upregulated in the female preterm with diabetes compared to both the term nondiabetic and term diabetic groups. Further, there was significant enrichment of Notch signalling‐related GO terms in preterm mice with diabetes of both sexes compared to term mice with diabetes, indicating activation of this pathway as a shared feature across sexes and increased Notch signalling appears to be attributable to the preterm birth. This is interesting because in early life, the receptors *Notch1* and *2* are key players in differentiation of the proximal nephron and podocytes [78], whereas *Notch3* and *4* contribute to vascular development [79, 80, 81, 82]. However, during DKD, upregulation of Notch signalling is a key mediator of podocyte injury, fibrosis, and inflammation [83, 84, 85]. Its inhibition is protective in experimental models with male mice and rats [84]. However, while *Notch1* and *Notch3* were upregulated in PT‐D males, they did not meet the threshold for differential expression, suggesting a potential subtler response compared to females.

Sex differences in the progression of DKD have been previously reported. Several studies suggest that male mice are more susceptible to kidney injury and alterations than females. In a hyperglycemia model using multiple mouse strains treated with low‐dose STZ, female mice exhibited less severe kidney injury and structural alterations, while male mice developed more pronounced hyperglycemia and injury [86]. Similarly, a study using a genetic model of diabetes with Ins2^Akita^ mice demonstrated more severe DKD in males compared to females [87]. Our findings are consistent with these studies; we observed a more mild effect in female mice with less severe kidney injury compared to males. In our model, podocyte density was similar between preterm diabetic females and term diabetic females. However, preterm diabetic male mice had a significantly lower podocyte density relative to term diabetic males. Podocyte injury and loss are central drivers of DKD progression [88], and preterm birth can result in a reduction in the number of functional podocytes or greater podocyte loss as shown in human and animal studies [21, 89, 90, 91]. The podocyte depletion hypothesis links preterm birth to chronic kidney disease by proposing that progressive podocyte loss drives proteinuria, glomerulosclerosis, and eventual kidney failure [92, 93]. Developmental deficits or acquired injury of podocytes can accelerate this process. Females appear relatively protected, likely due to higher baseline podocyte density and reduced susceptibility to podocyte loss, providing greater functional reserve. This protection may mitigate the combined renal stress of preterm birth and hyperglycemia. Further, there was no difference in renal *Ren1* expression between preterm diabetic females and term diabetic females, but there was a significant difference in males. This suggests that the impact of diabetes on the renin–angiotensin system may be sex‐specific, and female mice may have relatively preserved renin expression despite preterm birth. Previous studies have highlighted sex differences in the regulation of the renin–angiotensin system, and our findings are consistent with a more attenuated response in females [17, 94]. Females also appear resistant to reduced renin expression due to exposure to hyperglycemia alone, unlike males, potentially indicating protective mechanisms that mitigate the influence of diabetes on juxtaglomerular renin release in females [95].

### 4.1 Limitations

There are several limitations to this study. First, the preterm mice were delivered 1 day early. Our previous work has suggested that mice born 2 days preterm correspond approximately to a human gestational age of 27 weeks, indicating that our cohort models moderate preterm birth. Unlike humans, who typically complete nephrogenesis at full term, mice continue to generate nephrons for 4 days postnatally [96]. Nonetheless, both animal studies and autopsy studies of preterm human neonates indicate truncated nephrogenesis following early delivery, supporting the use of mouse models to investigate the effects of preterm birth on kidney development despite these species‐specific differences [6, 8, 21]. Second, there are limitations to the interpretation of some of the differentially expressed gene pathways. Without further experiments beyond the scope of this project, we have used the published literature to interpret the pathway or genes changes to (1) be signs of greater injury if they are known to be upregulated in DKD and promote injury when upregulated or (2) an attempt at protection when the pathways are normally downregulated in DKD, and when activated are protective. We acknowledge post translational modifications of these proteins may play a role. The lack of blood pressure is a limitation. In addition, the relatively modest sample size limits the statistical power, particularly for pathways with modest effect sizes or variable expression across groups.

STZ‐induced hyperglycemia is a variable model of diabetes, and the literature suggests that female mice are resistant to STZ‐induced hyperglycemia [19, 24]. Although high doses typically induce a more severe hyperglycemia [20], a low‐dose protocol was used here due to its more favourable mortality profile. As recommended by the Animal Models of Diabetic Complications Consortium, redosing is often required in the low‐dose model because a single treatment course frequently fails to produce a blood glucose level > 300 mg/dL. Several mice required a second round of STZ treatment (T‐D male, *n* = 1; PT‐D male, *n* = 2; PT‐D female, *n* = 3). Despite the need for redosing and a potentially shorter window of exposure to hyperglycemia, PT male and female mice had evidence of more severe DKD. However, because animals that required repeated STZ dosing were included in the analysis, this likely contributed, at least in part, to the modest phenotype observed in the females. Limited sample size and abbreviated hyperglycemia exposure resulting from repeated STZ dosing may have reduced the ability to detect significant functional, structural, and transcriptome differences between PT‐D and either T‐ND or T‐D, including GFR. Additionally, sex differences are therefore difficult to fully separate from differences in STZ responsiveness, with females exhibiting a milder diabetic phenotype consistent with prior reports of reduced β‐cell susceptibility [97]. Nonetheless, these sex differences remain biologically important because they likely contribute to the differential outcomes observed and may reflect underlying mechanisms relevant to both diabetes and preterm birth. As noted in our previous report [18], this model primarily reflects type 1 diabetes and thus does not fully capture the broader population of individuals born preterm who later develop diabetes because these individuals born preterm are at increased risk for both type 1 and type 2 diabetes. Preterm birth disrupts β‐cell development, which occurs predominantly in late gestation, potentially predisposing these individuals to an early type 1 diabetes phenotype characterized by reduced insulin production, in addition to insulin resistance. Alternative models would be valuable for characterizing the effects of a more type 2 diabetes–like phenotype.

## 5 Conclusions

Preterm birth increases vulnerability to hyperglycemia‐induced kidney injury in female mice. Although structural changes in the kidneys of female mice are mild at this early stage of DKD, the molecular alterations observed appear to occur early and act as initial drivers of disease progression. Interestingly, preterm females seem to show relative protection against kidney injury, suggesting sex‐specific mechanisms that may influence susceptibility to DKD progression. These results highlight the critical importance of including both sexes in research since understanding protective mechanisms in one sex may also inform new strategies to reduce risk in a more susceptible group. Moreover, while advances in neonatal care continue to lower the gestational age of viability, a growing population of individuals born extremely preterm are now surviving beyond infancy and will develop chronic disease such as diabetes. Our findings underscore the need for a deeper understanding of the mechanisms that drive the progression of kidney disease in those born preterm and support the development of targeted monitoring and personalized management strategies for these individuals.

## Author Contributions

**Ayyappa Kumar Sista Kameshwar:** formal analysis, visualization, writing – review and editing. **Rachel K. Dailey:** data curation, formal analysis, visualization, writing – original draft, methodology, investigation, project administration, writing – review and editing, resources, validation. **Masako Suzuki:** formal analysis, visualization, methodology, supervision, writing – review and editing, validation. **Logan C. Hamil:** investigation, writing – review and editing. **Kevin M. Bennett:** formal analysis, writing – review and editing, investigation. **Sage Timberline:** writing – review and editing. **Jaya Isaac:** investigation, writing – review and editing. **Kimberly deRonde:** conceptualization, investigation, writing – review and editing. **Edwin J. Baldelomar:** formal analysis, investigation, writing – review and editing. **Aleksandra Cwiek:** conceptualization, investigation, methodology, writing – review and editing. **Matthew R. Hoch:** investigation, writing – review and editing. **Mark Conaway:** formal analysis, writing – review and editing. **Kimberly J. Reidy:** investigation, formal analysis, supervision, writing – review and editing. **Jennifer R. Charlton:** conceptualization, data curation, formal analysis, visualization, writing – original draft, writing – review and editing, project administration, supervision, investigation, methodology, validation, funding acquisition, resources.

## Funding

J.R.C., M.S., K.J.R.: R01DK136989, J.R.C.: 5U24DK115255‐04 (diabetic consortium complications); J.R.C. (indirect): P50DK096373‐11. Imaging data were acquired through the University of Virginia Molecular Imaging Core Laboratory with National Institutes of Health S10OD025024 funding.

## Disclosure

J.R.C.: co‐owner of Sindri Technologies LLC, investor in ZorroFlow, consultant for Mozarc Medical, speaker for Mead Johnson Nutrition, President of Neonatal Kidney Collaborative; K.M.B.: co‐owner of Sindri Technologies LLC, co‐owner of XN Biotechnologies LLC; E.J.B.: co‐owner of XN Biotechnologies LLC.

## Conflicts of Interest

The authors declare no conflicts of interest.

## Supporting information


**Figure S1:** RNA Quality Assessment (a) RNA integrity number (RIN), (b) uniquely mapped reads, (c) RNA concentration, and (d) A260/A280 ratios did not differ between groups. (e) Additionally, no correlations were observed between RIN and uniquely mapped reads (f) or between RIN and duplication percentage.
**Figure S2:** Characterization of the females in the model. (a) Diabetic females weighed less than non‐diabetic females 12 weeks after initial STZ treatment. (b) Term diabetic (T‐D) and preterm diabetic (PT‐D) females weighed less than non‐diabetic females (T‐ND and PT‐ND) prior to euthanasia. (c) STZ successfully induced hyperglycemia in term and preterm females. (d) T‐D and PT‐D females had a greater blood glucose level than T‐ND females prior to euthanasia. T‐D had a greater blood glucose level than T‐ND, and PT‐D had a greater blood glucose level than PT‐ND. (e) The kidneys from T‐D females weighed more than the kidneys of T‐ND females. (f) The diabetic females (T‐D and PT‐D) had a greater kidney weight to body weight ratio compared to T‐ND. (g) There was no difference in glomerular filtration rate (GFR) across all animals. (h) The urine albumin to creatinine ratio (urine ACR) was greater in PT‐D compared to T‐ND. All experimental groups had urine ACR within the normal range (urine ACR < 30 mg/g; indicated with dotted line). (i) Blood urea nitrogen (BUN) level was greater in PT‐D than both T‐D and PT‐ND. BUN level was lower in T‐D than T‐ND. (j) Glomerular density was not statistically different between female animals. Two‐tailed Mann–Whitney tests (a, b, c, d, e, f, g, i, j), one‐tailed Mann–Whitney tests (h), with a *p*‐value < 0.05 considered statistically significant.
**Figure S3:** Histological assessments. (a) Percentage of renin positive glomeruli was similar between T‐D and PT‐D. PT‐D had more renin positive glomeruli than PT‐ND. (b) Podocyte density was lower in PT‐D compared to PT‐ND (c) There was no difference in glomerular number by the stereologic method, Weibel‐Gomez. (d) There was no difference in average glomerular area between groups, based on the mean area of glomeruli measured from segmented slides used to estimate glomerular number by stereology. Two‐tailed Mann–Whitney tests (a, b, c, d) with a *p*‐value < 0.05 considered statistically significant.
**Figure S4:** Gene Ontology Analysis of Bulk RNA‐Seq for Differential Expressed Genes Between T‐ND and PT‐D: Molecular Function GO terms. (a) GO enrichment analysis revealed enrichment of the GO terms “cell adhesion molecule binding” and “extracellular matrix structural constituent” in PT‐D upregulated genes compared to T‐ND. (b) Transporter activity‐related GO terms were enriched in genes downregulated in PT‐D relative to T‐ND. (c, d) CNET plots display the connections between GO terms and their associated genes.
**Figure S5:** qRT‐PCR validation of bulk RNA‐seq analysis. qRT‐PCR analysis demonstrated increased expression of (a) *Hmox1*, (b) *Nos3*, and (c) *Notch1* in preterm diabetic (PT‐D) kidney compared with term diabetic (T‐D) kidney.
**Figure S6:** Gene Ontology Analyses of Bulk RNA‐Seq for Differential Expressed Genes Between T‐D and PT‐D: Molecular Function GO terms. (a) The GO term “Notch binding” was enriched in upregulated genes in PT‐D compared to T‐D. (b) The GO term “structural constituent of ribosomes” was the only enrichened pathway in PT‐D downregulated genes relative to T‐D. (c, d) CNET plots display the connections between GO terms and their associated genes.
**Figure S7:** Secondary bulk RNA‐seq volcano plots. (a) Differential gene expression was assessed between term non‐diabetic (T‐ND) and term diabetic (T‐D) groups, (b) as well as between preterm non‐diabetic (PT‐ND) and preterm diabetic (PT‐D) groups, to evaluate baseline transcriptional differences associated with diabetes status within term and preterm animals.
**Figure S8:** Further characterization of sex differences in the model. (a) Female animals weighed less than male animals across all conditions. T‐D and PT‐D mice had similar body weight at 18 weeks in both males and females (b) Several males required a second round of STZ treatment (T‐D male, *n* = 1; PT‐D male, *n* = 2). Both PT‐D males that received a second round of STZ reached terminal glucose levels exceeding 300 mg/dL, whereas the T‐D male remained below 300 mg/dL following repeat STZ treatment. T‐D males and T‐D females had similar terminal blood glucose levels. Terminal blood glucose level was lower in preterm diabetic females than preterm diabetic males. For males, the average blood glucose of the T‐D was 523 ± 109 mg/dL (T‐ND: 130 ± 28 mg/dL) and PT‐D was 535 ± 159 mg/dL (P‐ND: 140 ± 42 mg/dL). For females, the average blood glucose of the T‐D was 441 ± 74 mg/dL (T‐ND: 121 ± 25 mg/dL) and PT‐D was 377 ± 184 mg/dL (P‐ND: 107 ± 12 mg/dL). (c) Male animals across all conditions had heavier kidneys than females. (d) There were no sex differences in GFR, (e) serum urea nitrogen, (f) urine albumin to creatinine ratio, (g) renin positive glomeruli, and (h) glomerular number by stereology across any condition male PT‐D: 12386 (11965–13,978) vs. female PT‐D: 13911 (11177–15,046). Two‐tailed Mann–Whitney tests with a *p*‐value < 0.05 considered statistically significant.
**Figure S9:** Sex‐stratified principal component analysis across term and preterm non‐diabetic and diabetic groups. Principal component analysis (PCA) of male and female non‐diabetic mice (T‐ND and PT‐ND) and diabetic mice (T‐D and PT‐D) revealed a distinction between term and preterm samples, with the exception of T‐ND males.
**Figure S10:** Gene Ontology Analyses of Bulk RNA‐Seq for Differential Expressed Genes Between PT‐D and T‐D Across Both Sexes. (a) The enrichment of GO terms “Notch signaling pathway”, “negative regulation of Notch signaling pathway”, and (b) “Notch binding” in downregulated genes in PT‐D compared to T‐D. (c, d) The linkages of corresponding genes and those terms are shown in the CNET plots. (a, c) GO enriched terms: biological processes and (b, d) molecular functions.

**Table S1:** Reagents and resources.
**Table S2:** Bulk RNA‐seq sequencing statistics.
**Table S3:** qRT‐PCR Primers.
**Table S4:** A list of DEGs between T‐ND and PT‐D females.
**Table S5:** A list of DEGs between T‐D and PT‐D females.
**Table S6:** A list of DEGs between T‐ND and T‐D females.
**Table S7:** A list of DEGs between PT‐ND and PT‐D females.

## Data Availability

The data that support the findings of this study are openly available in GEO at https://www.ncbi.nlm.nih.gov/geo/, reference number GSE319377.
