# Supplementary material for: Preterm Birth Increases Susceptibility to Hyperglycemia‐Induced Kidney Injury With Sex‐Specific Differences in Structural and Molecular Responses
Source: Endocrinol Diabetes Metab. 2026 Apr 20;9(3):e70223. doi: 10.1002/edm2.70223 (PMC13095165; doi:10.1002/edm2.70223)
Supplement: Supplementary file 1 — Figure S1: RNA Quality Assessment (a) RNA integrity number (RIN), (b) uniquely mapped reads, (c) RNA concentration, and (d) A260/A280 ratios did not differ between groups. (e) Additionally, no correlations were observed between RIN and uniquely mapped reads (f) or between RIN and duplication percentage. Figure S2: Characterization of the females in the model. (a) Diabetic females weighed less than non‐diabetic females 12 weeks after initial STZ treatment. (b) Term diabetic (T‐D) and preterm diabetic (PT‐D) females weighed less than non‐diabetic females (T‐ND and PT‐ND) prior to euthanasia. (c) STZ successfully induced hyperglycemia in term and preterm females. (d) T‐D and PT‐D females had a greater blood glucose level than T‐ND females prior to euthanasia. T‐D had a greater blood glucose level than T‐ND, and PT‐D had a greater blood glucose level than PT‐ND. (e) The kidneys from T‐D females weighed more than the kidneys of T‐ND females. (f) The diabetic females (T‐D and PT‐D) had a greater kidney weight to body weight ratio compared to T‐ND. (g) There was no difference in glomerular filtration rate (GFR) across all animals. (h) The urine albumin to creatinine ratio (urine ACR) was greater in PT‐D compared to T‐ND. All experimental groups had urine ACR within the normal range (urine ACR < 30 mg/g; indicated with dotted line). (i) Blood urea nitrogen (BUN) level was greater in PT‐D than both T‐D and PT‐ND. BUN level was lower in T‐D than T‐ND. (j) Glomerular density was not statistically different between female animals. Two‐tailed Mann–Whitney tests (a, b, c, d, e, f, g, i, j), one‐tailed Mann–Whitney tests (h), with a p‐value < 0.05 considered statistically significant. Figure S3: Histological assessments. (a) Percentage of renin positive glomeruli was similar between T‐D and PT‐D. PT‐D had more renin positive glomeruli than PT‐ND. (b) Podocyte density was lower in PT‐D compared to PT‐ND (c) There was no difference in glomerular number by the stereologic method, Wei [file EDM2-9-e70223-s001.docx]

**Supplementary Figures**

**Preterm Birth Increases Susceptibility to Hyperglycemia-Induced Kidney Injury with Sex-Specific Differences in Structural and Molecular Responses**

Rachel K Dailey^1^, Aleksandra Cwiek^2^, Logan C Hamil^3^, Sage Timberline^4^, Ayyappa Kumar Sista Kameshwar^5^, Masako Suzuki^5^, Jaya Isaac^6^, Kimberly deRonde^1^, Mark Conaway^7.8^, Kevin M Bennett^9^, Edwin J Baldelomar^9^, Matthew R Hoch^10^, Kimberly J Reidy^6^, Jennifer R Charlton^1*^

***sFigure 1. RNA Quality Assessment (a)*** *RNA integrity number (RIN),* ***(b)*** *uniquely mapped reads,* ***(c)*** *RNA concentration, and* ***(d)*** *A260/A280 ratios did not differ between groups.* ***(e)*** *Additionally, no correlations were observed between RIN and uniquely mapped reads* ***(f)*** *or between RIN and duplication percentage.*

*
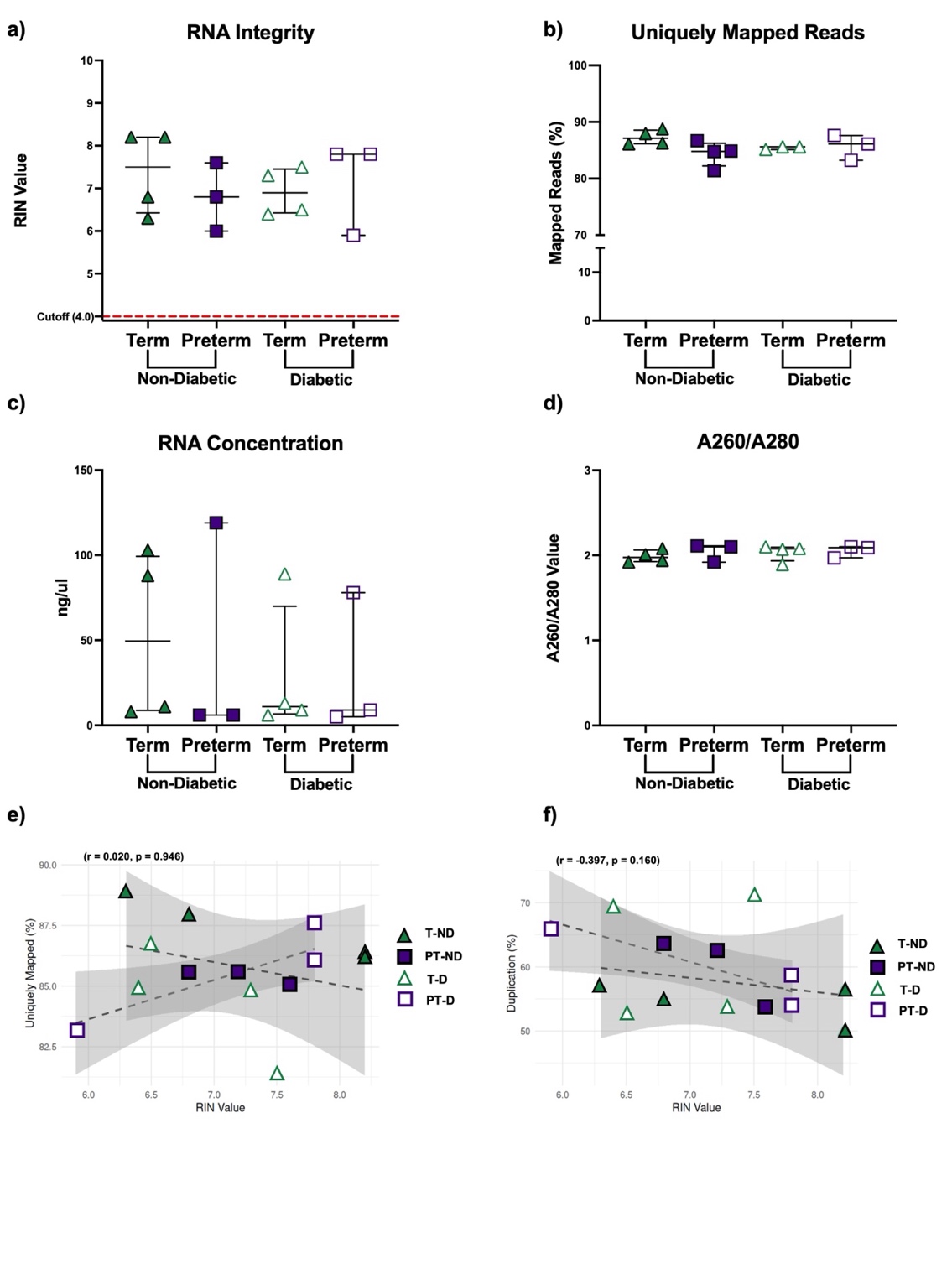
*

***sFigure 2. Characterization of the females in the model. (a)*** *Diabetic females weighed less than non-diabetic females 12 weeks after initial STZ treatment.* ***(b)*** *Term diabetic (T-D) and preterm diabetic (PT-D) females weighed less than non-diabetic females (T-ND and PT-ND) prior to euthanasia.* ***(c)*** *STZ successfully induced hyperglycemia in term and preterm females.* ***(d)*** *T-D and PT-D females had a greater blood glucose level than T-ND females prior to euthanasia. T-D had a greater blood glucose level than T-ND, and PT-D had a greater blood glucose level than PT-ND.* ***(e)*** *The kidneys from T-D females weighed more than the kidneys of T-ND females.* ***(f)*** *The diabetic females (T-D and PT-D) had a greater kidney weight to body weight ratio compared to T-ND.* ***(g)*** *There was no difference in glomerular filtration rate (GFR) across all animals.* ***(h)*** *The urine albumin to creatinine ratio (urine ACR) was greater in PT-D compared to T-ND. All experimental groups had urine ACR within the normal range (urine ACR<30 mg/g; indicated with dotted line).* ***(i)*** *Blood urea nitrogen (BUN) level was greater in PT-D than both T-D and PT-ND. BUN level was lower in T-D than T-ND.* ***(j)*** *Glomerular density was not statistically different between female animals.* *Two-tailed Mann-Whitney tests (a, b, c, d, e, f, g, i, j), one-tailed Mann-Whitney tests (h), with a p-value < 0.05 considered statistically significant.*

*
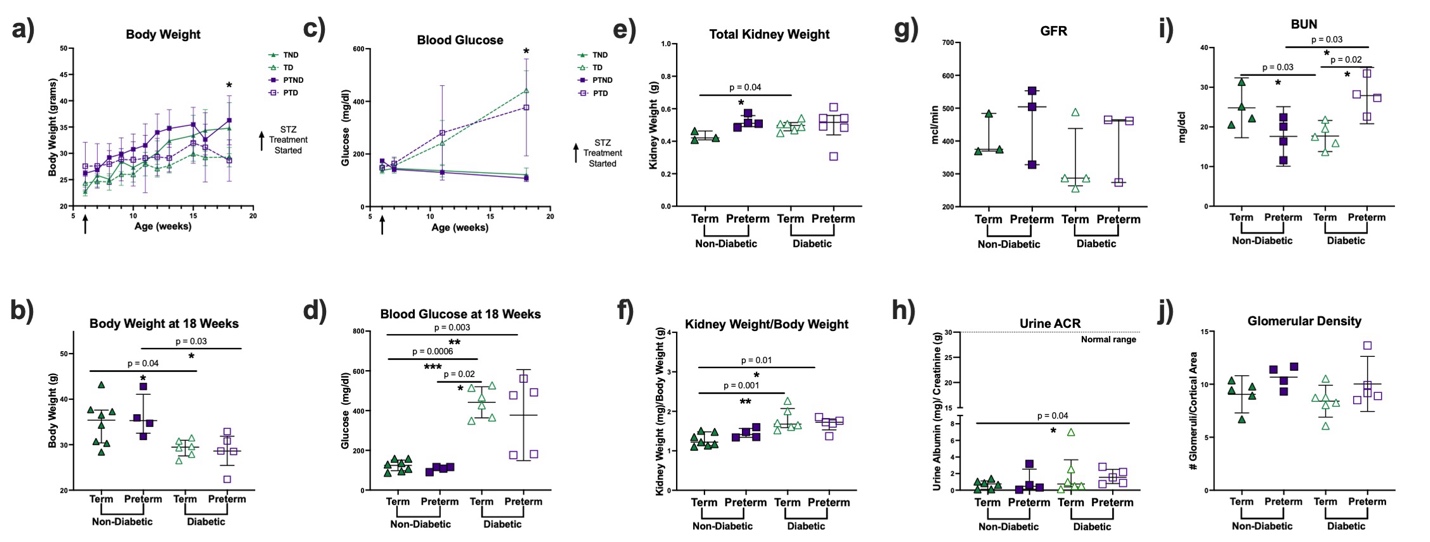
*

***sFigure 3. Histological assessments. (a)*** *Percentage of renin positive glomeruli was similar between T-D and PT-D. PT-D had more renin positive glomeruli than PT-ND.* ***(b)*** *Podocyte density was lower in PT-D compared to PT-ND* ***(c)*** *There was no difference in glomerular number by the stereologic method, Weibel-Gomez.* ***(d)*** *There was no difference in average glomerular area between groups, based on the mean area of glomeruli measured from segmented slides used to estimate glomerular number by stereology. Two-tailed Mann-Whitney tests (a, b, c, d) with a p-value < 0.05 considered statistically significant.*

*
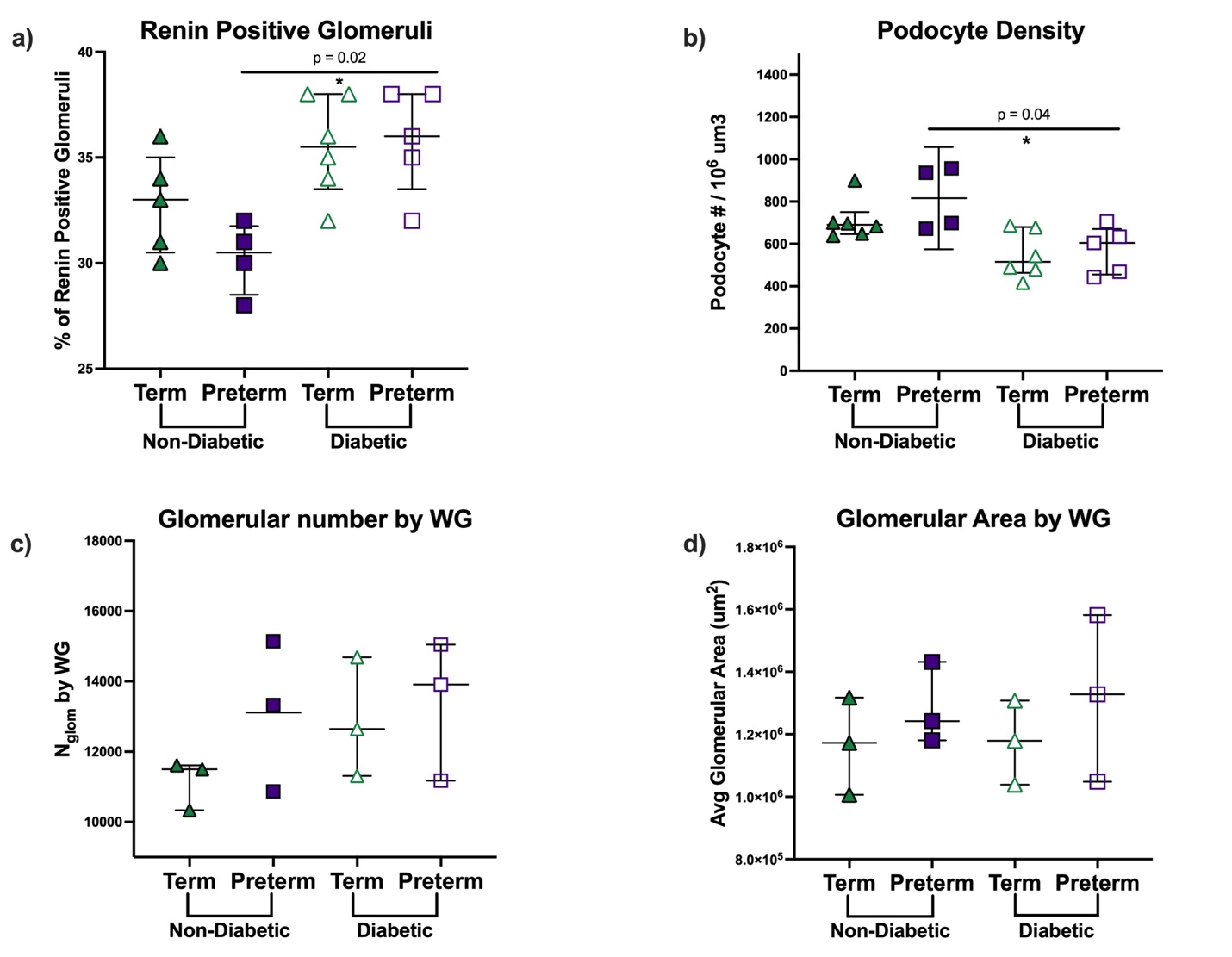
*

***sFigure 4. Gene Ontology Analysis of Bulk RNA-Seq for Differential Expressed Genes Between T-ND and PT-D: Molecular Function GO terms. (a)*** *GO enrichment analysis revealed enrichment of the GO terms “cell adhesion molecule binding” and “extracellular matrix structural constituent” in PT-D upregulated genes compared to T-ND.* ***(b)*** *Transporter activity-related GO terms were enriched in genes downregulated in PT-D relative to T-ND.* ***(c, d)*** *CNET plots display the connections between GO terms and their associated genes.*

*
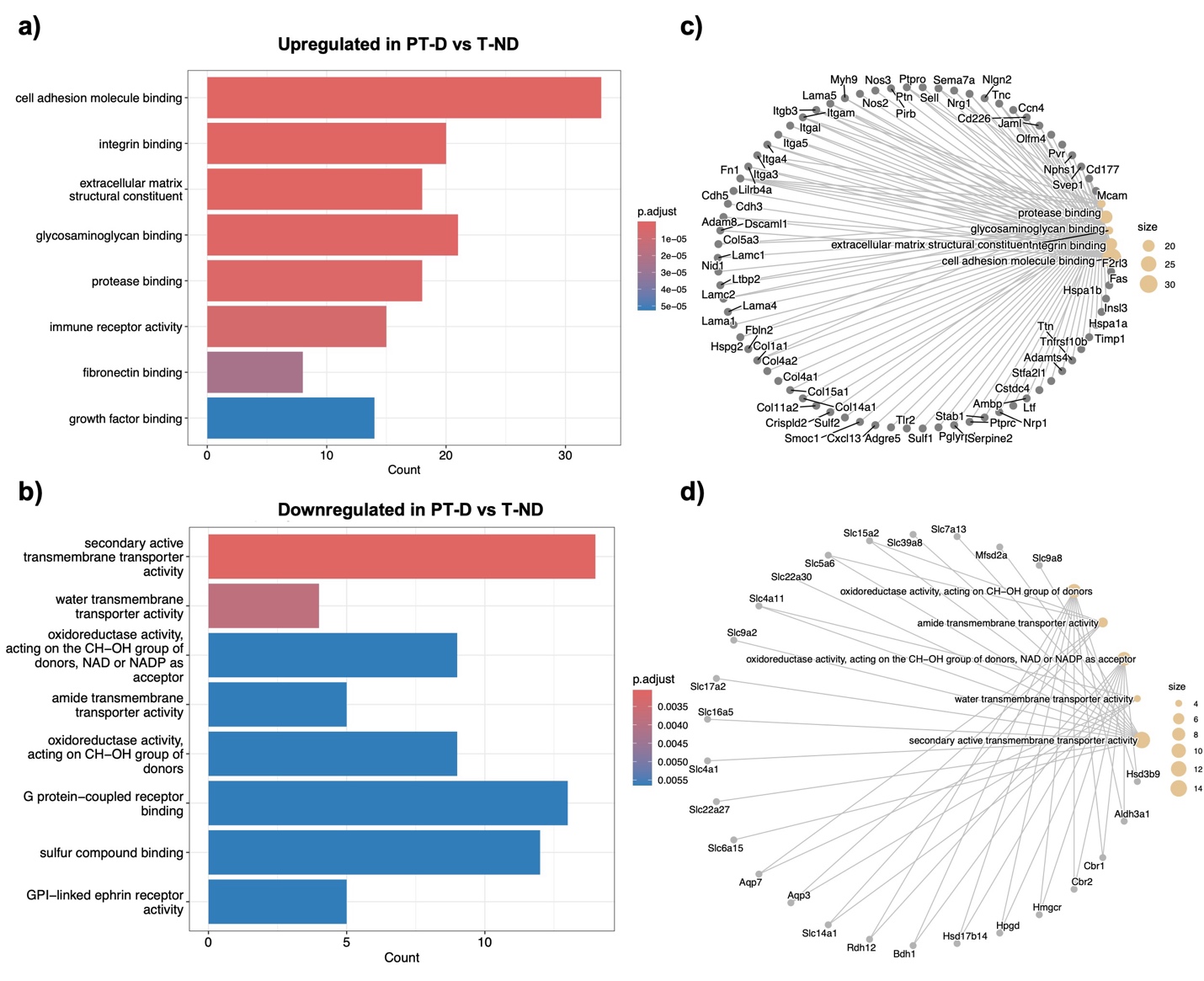
*

***sFigure 5. qRT-PCR validation of bulk RNA-seq analysis.*** *qRT-PCR analysis demonstrated increased expression of* ***(a)*** Hmox1*,* ***(b)*** Nos3*, and* ***(c)*** Notch1 *in preterm diabetic (PT-D) kidney compared with term diabetic (T-D) kidney.*

*
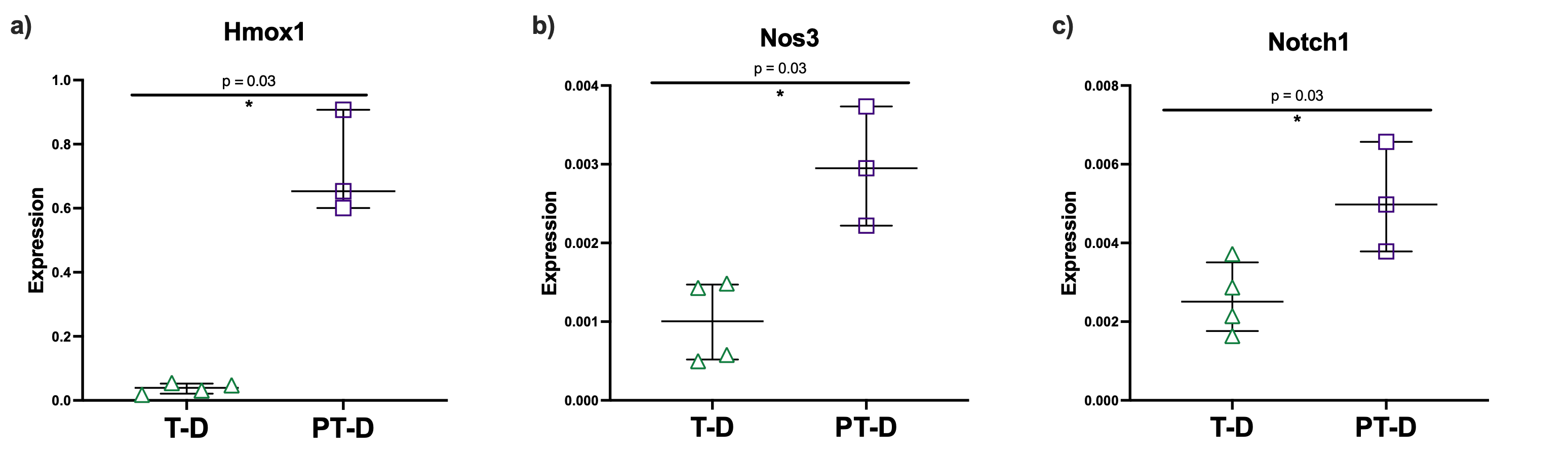
*

***sFigure 6. Gene Ontology Analyses of Bulk RNA-Seq for Differential Expressed Genes Between T-D and PT-D: Molecular Function GO terms. (a)*** *The GO term “Notch binding” was enriched in upregulated genes in PT-D compared to T-D.* ***(b)*** *The GO term “structural constituent of ribosomes” was the only enrichened pathway in PT-D downregulated genes relative to T-D.* ***(c, d)*** *CNET plots display the connections between GO terms and their associated genes.*

*
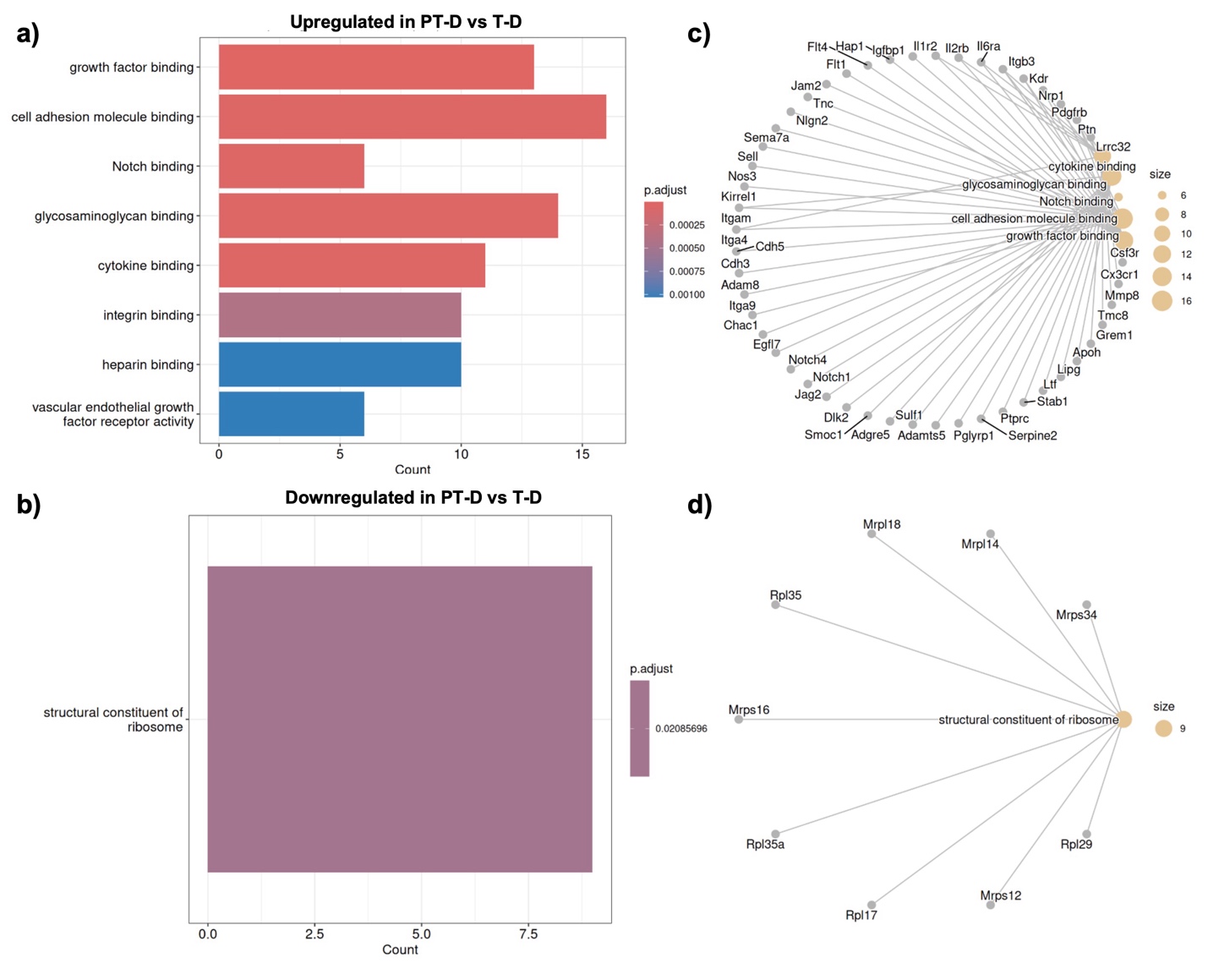
*

***sFigure 7. Secondary bulk RNA-seq volcano plots. (a)*** *Differential gene expression was assessed between term non-diabetic (T-ND) and term diabetic (T-D) groups,* ***(b)*** *as well as between preterm non-diabetic (PT-ND) and preterm diabetic (PT-D) groups, to evaluate baseline transcriptional differences associated with diabetes status within term and preterm animals.*

*
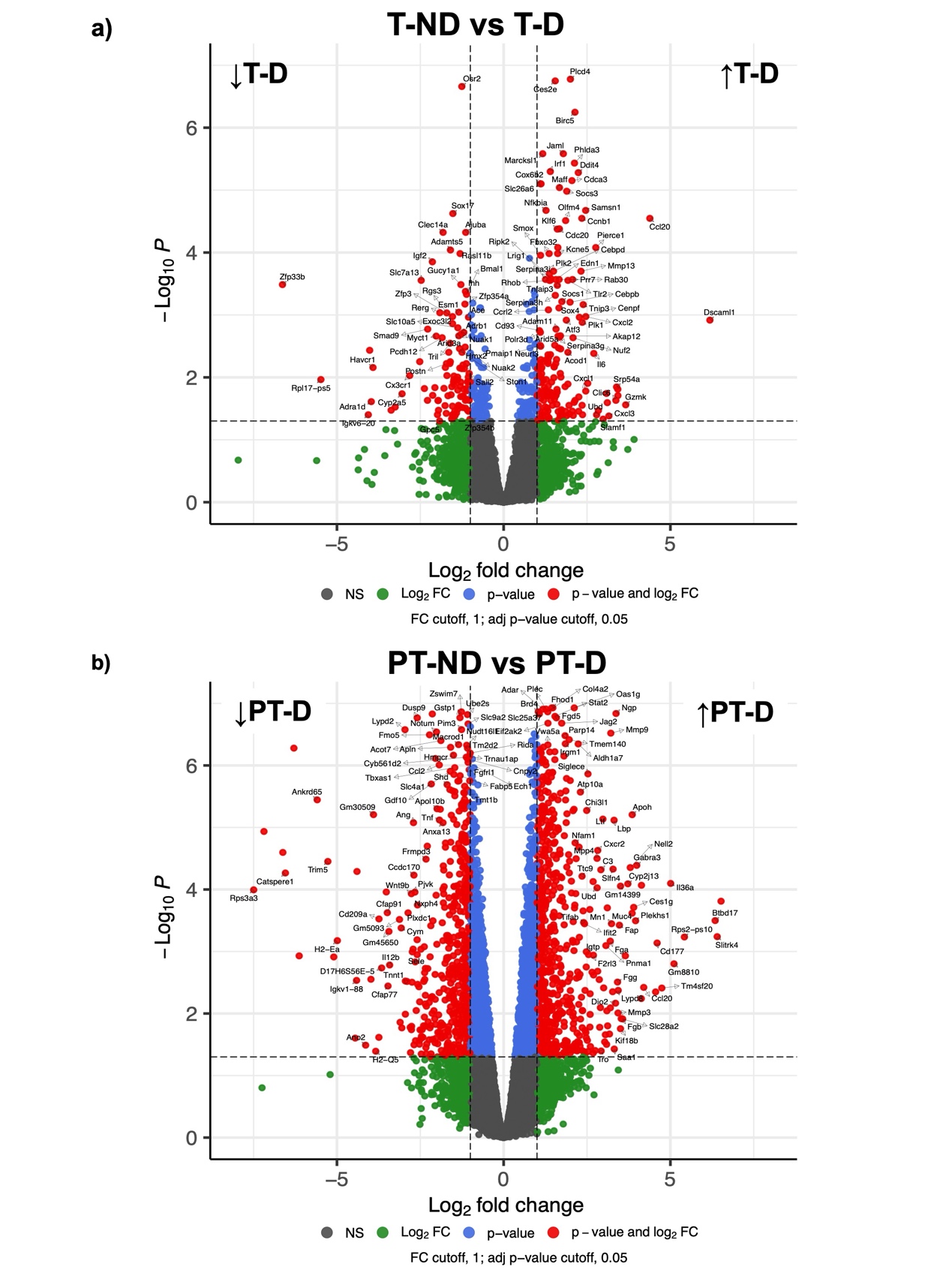
*

***sFigure 8. Further characterization of sex differences in the model.*** ***(a)*** *Female animals weighed less than male animals across all conditions. T-D and PT-D mice had similar body weight at 18 weeks in both males and females* ***(b)*** *Several males required a second round of STZ treatment (T-D male, n=1; PT-D male, n=2). Both PT-D males that received a second round of STZ reached terminal glucose levels exceeding 300 mg/dL, whereas the T-D male remained below 300 mg/dL following repeat STZ treatment. T-D males and T-D females had similar terminal blood glucose levels. Terminal blood glucose level was lower in preterm diabetic females than preterm diabetic males. For males, the average blood glucose of the T-D was 523 ± 109 mg/dl (T-ND: 130 ± 28 mg/dl) and PT-D was 535 ± 159 mg/dl (P-ND: 140 ± 42 mg/dl). For females, the average blood glucose of the T-D was 441± 74 mg/dl (T-ND: 121± 25 mg/dl) and PT-D was 377 ± 184 mg/dl (P-ND: 107 ± 12 mg/dl).* ***(c)*** *Male animals across all conditions had heavier kidneys than females.* ***(d)*** *There were no sex differences in GFR,* ***(e)*** *serum urea nitrogen,* ***(f)*** *urine albumin to creatinine ratio,* ***(g)*** *renin positive glomeruli, and* ***(h)*** *glomerular number by stereology across any condition (male PT-D: 12386 (11965-13978) vs female PT-D: 13911 (11177-15046). Two-tailed Mann-Whitney tests with a p-value < 0.05 considered statistically significant.*

*
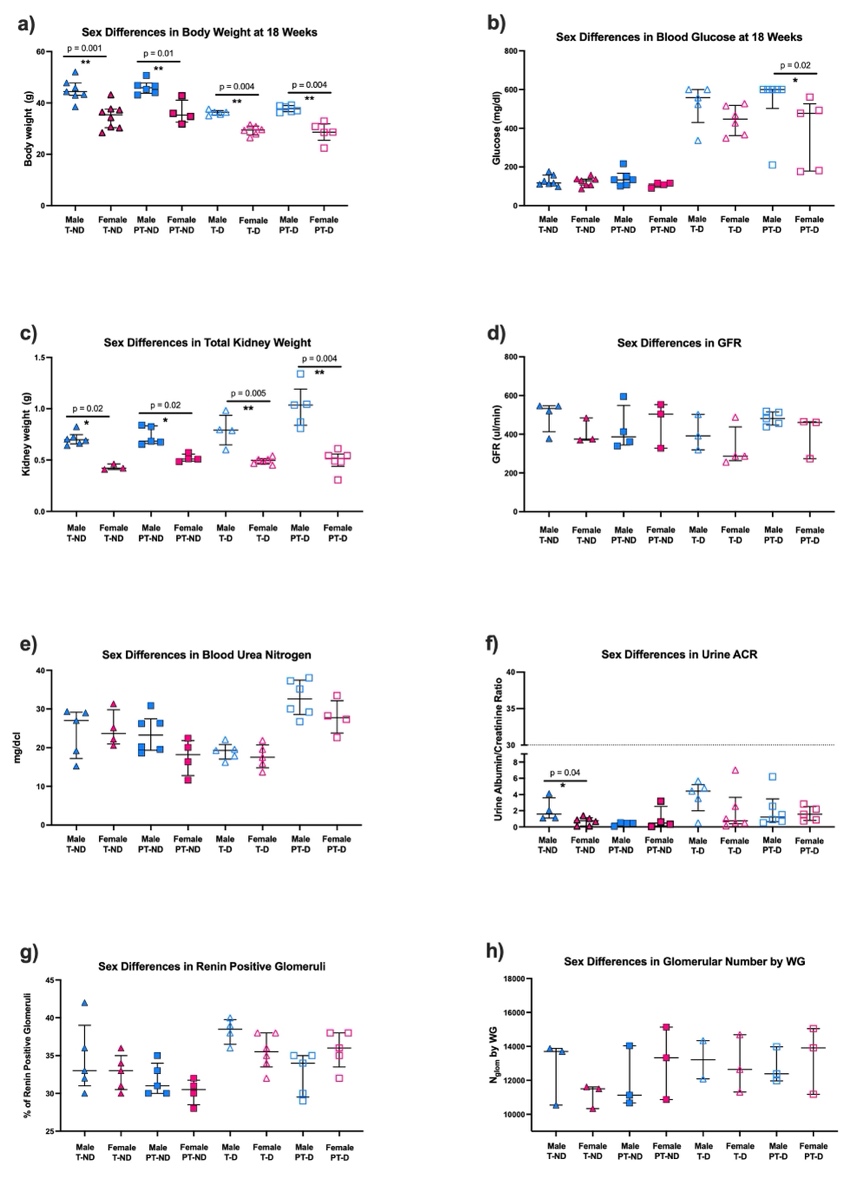
*

***sFigure 9. Sex-stratified principal component analysis across term and preterm non-diabetic and diabetic groups. (a)*** *Principal component analysis (PCA) of male and female non-diabetic mice (T-ND and PT-ND) and diabetic mice (T-D and PT-D) revealed a distinction between term and preterm samples, with the exception of T-ND males.*

*
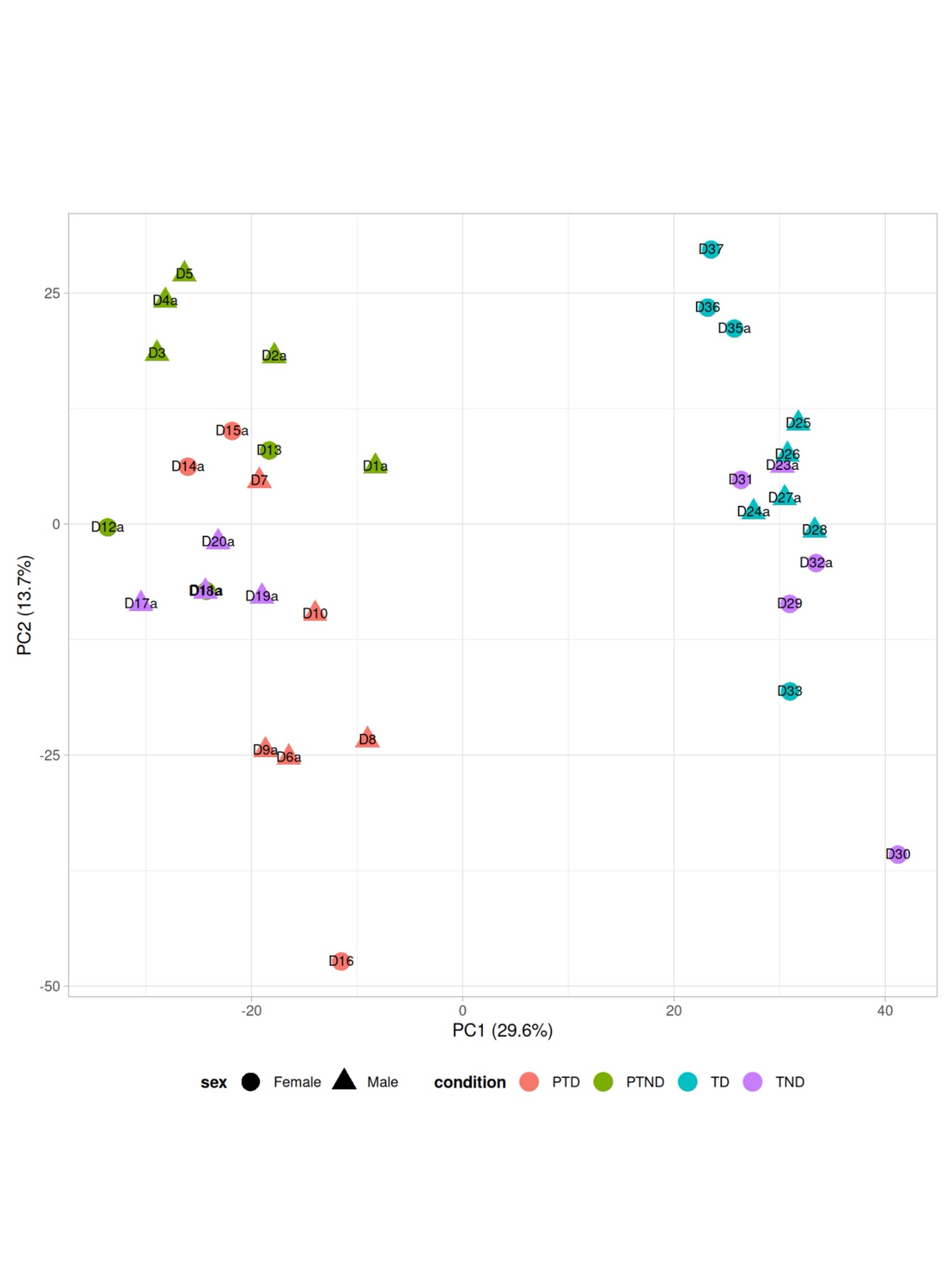
*

***sFigure 10****.* ***Gene Ontology Analyses of Bulk RNA-Seq for Differential Expressed Genes Between PT-D and T-D Across Both Sexes. (a)*** *The enrichment of GO terms “Notch signaling pathway”, “negative regulation of Notch signaling pathway”, and* ***(b)*** *“Notch binding”* *in downregulated genes in PT-D compared to T-D.* ***(c, d)*** *The linkages of corresponding genes and those terms are shown in the CNET plots.* ***(a, c)*** *GO enriched terms: biological processes and* ***(b, d)*** *molecular functions.*

*
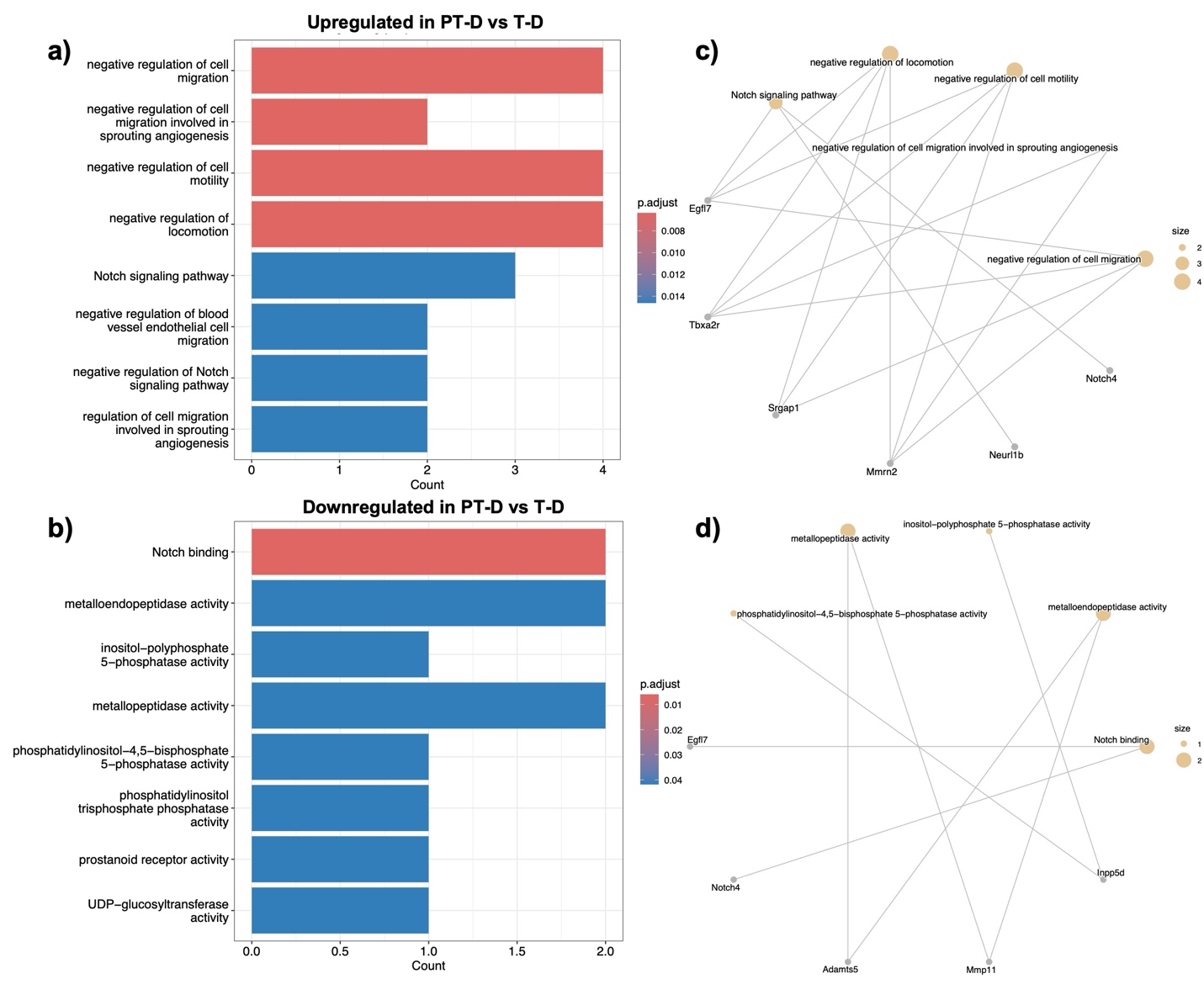
*
